# Early activation of deleterious molecular pathways in the kidney in experimental heart failure with atrial remodeling

**DOI:** 10.14814/phy2.13283

**Published:** 2017-05-15

**Authors:** Tomoko Ichiki, Brenda K. Huntley, Gail J. Harty, S. Jeson Sangaralingham, John C. Burnett

**Affiliations:** ^1^ Cardiorenal Research LaboratoryDepartment of Cardiovascular MedicineMayo ClinicRochesterMinnesota

**Keywords:** Heart failure, inflammation, kidney, natriuretic peptides

## Abstract

Heart failure (HF) is a major health problem with worsening outcomes when renal impairment is present. Therapeutics for early phase HF may be effective for cardiorenal protection, however the detailed characteristics of the kidney in early‐stage HF (ES‐HF), and therefore treatment for potential renal protection, are poorly defined. We sought to determine the gene and protein expression profiles of specific maladaptive pathways of ES‐HF in the kidney and heart. Experimental canine ES‐HF, characterized by de‐novo HF with atrial remodeling but not ventricular fibrosis, was induced by right ventricular pacing for 10 days. Kidney cortex (KC), medulla (KM), left ventricle (LV), and left atrial (LA) tissues from ES‐HF versus normal canines (*n* = 4 of each) were analyzed using RT‐PCR microarrays and protein assays to assess genes and proteins related to inflammation, renal injury, apoptosis, and fibrosis. ES‐HF was characterized by increased circulating natriuretic peptides and components of the renin‐angiotensin‐aldosterone system and decreased sodium and water excretion with mild renal injury and up‐regulation of CNP and renin genes in the kidney. Compared to normals, widespread genes, especially genes of the inflammatory pathways, were up‐regulated in KC similar to increases seen in LA. Protein expressions related to inflammatory cytokines were also augmented in the KC. Gene and protein changes were less prominent in the LV and KM. The ES‐HF displayed mild renal injury with widespread gene changes and increased inflammatory cytokines. These changes may provide important clues into the pathophysiology of ES‐HF and for therapeutic molecular targets in the kidney of ES‐HF.

## Introduction

The kidney plays a key role in heart failure (HF) exemplified by the fact that impaired renal function increases the risk for poor outcomes. Studies support the concept that reduced myocardial pump function with either reduced arterial pressure and/or increased venous pressure in HF impairs renal perfusion and induces sodium and water retention which may result in congestion and diuretic resistance (Mullens et al. 2009). Further, meta‐analysis of 85 clinical studies of renal impairment in patients with HF reported that worsening renal function was prevalent and strongly associated with increased mortality risk (Damman et al. 2014).

It is well established that progressive HF is mediated in part by activation of detrimental molecular pathways in the heart, even in the earliest stages of HF (Mann and Bristow 2005). Contributing to activation of such deleterious pathways is enhanced activity of the circulating and myocardial renin‐angiotensin‐aldosterone system (RAAS). The activation of RAAS may result in direct injury to the heart as well as serve to offset the protective myocardial actions of the natriuretic peptides (NPs) produced in the heart. Indeed, the recently successful HF therapeutic strategy using Entresto, an angiotensin receptor 1 blocker plus neprilysin inhibitor, is based upon simultaneously targeting both the RAAS and the NP system (Packer et al. 2015). Recently, Virzi and co‐workers reported that plasma from patients with acute HF caused increased gene expression of pro‐apoptotic and renal injury genes in cultured human renal tubular cells (Virzi et al. 2015). In addition, we have previously reported that in experimental HF, the highest levels of tissue Angiotensin II (ANG II) are found in the kidney cortex and medulla compared with lower levels in the atrial or ventricular myocardium (Luchner et al. 1996). This ANG II expression could activate the RAAS and other pathological renal molecular pathways leading to acute and chronic alterations in renal function and structure thus contributing to the progression of HF (Dries et al. 2000).

While the important role of the kidney in HF is acknowledged, the best timing to provide effective cardiorenal protective therapeutics has been incompletely explored. In other words, therapeutics for early phase HF may be more effective for cardiorenal protection; however, the detailed characteristics of the kidney in ES‐HF are poorly defined. We have reported that differential regulation of the natriuretic peptide (NP) system may contribute to atrial remodeling in ES‐HF (Ichiki et al. 2013). Here we expand our study, using an ES‐HF model with atrial fibrosis, to define in kidney cortex and medulla, changes in the NP and RAAS systems which may reflect the early evolving kidney changes relevant to human HF (Ichiki et al. 2013). Specifically, we defined changes in 179 selective genes related to inflammation, apoptosis, renal injury and fibrosis as well as components of the RAAS and NP system in the kidney as well as the heart. We hypothesized that gene changes in these pathways will occur in the kidney in ES‐HF, underscoring ES‐HF as a cardiorenal disease. Such findings may have significant implications for HF therapies, identifying both renal and myocardial targets for novel therapeutics.

## Materials and Methods

All animal experimental protocols used in the current study were approved by the Animal Care and Use Committee at Mayo Clinic.

### Normal and HF canines

Studies were conducted in 2 groups of male mongrel dogs (*n* = 4 for each group; weight, 20–28 kg), which included normal control and pacing‐induced experimental HF canines. HF was induced by rapid right ventricular pacing at 240 bpm as previously described and characterized which mimics de‐novo HF with early phase remodeling of atrium not ventricle (Boerrigter et al. 2007; Chen et al. 2009; Costello‐Boerrigter et al. 2010; Ichiki et al. 2013). In brief, all HF dogs underwent implantation of a programmable cardiac pacemaker (Medtronic, MN). After pre‐operative antibiotic treatment with cefazolin (40 mg/kg) and the induction of anesthesia using atropine (0.05 mg/kg, IM), ketamine (10–13 mg/kg, IV), Diazepam (0.5 mg/kg, IV), and morphine (0.5 mg/kg, IM), artificial ventilation (Harvard respirator, Harvard Apparatus, Millis, MA) with supplemental oxygen was started and a left lateral thoracotomy was performed under maintenance anesthesia with continuous IV infusion of morphine (0.24 mg/kg/h)/ketamine (0.6 mg/kg/h)/lidocanine (3 mg/kg/h) cocktail, isoflurane inhalation, and buvicaine injection as an intercostal nerve block. Via a 3–4 cm incision of pericardium, a screw‐in epicardial pacemaker lead was implanted into the right ventricle. The pacemaker generator was implanted subcutaneously into the left chest wall and connected to the pacemaker lead. Dogs received post‐operative prophylactic antibiotic treatment with cephalosporin (5–10 mg, PO) for 5 days and triple antibiotic ointment on the incision areas occasionally, and post‐operative analgesic treatment with buprenorphine SR (0.18 mg/kg, SQ) once immediately post operation and rimadyl (4 mg/kg, PO) once daily up to 5 days. Following a 14‐day post‐operative recovery period, the pacemaker was turned on at 240 bpm. Myocardial function and dimensions were assessed by echocardiography using a standard two‐dimensional echocardiogram on day 10. On day 11 of rapid ventricular pacing, the experiment was carried out under anesthesia with intravenous injection of sodium pentobarbital (Induction, 15 mg/kg; and maintenance 5–15 mg/kg/h) and fentanyl (Induction, 0.004–0.012 mg/kg; and maintenance 0.004–0.018 mg/kg/h), intubation, and mechanical ventilation with supplemental oxygen (Harvard respirator, MA) for determination of hemodynamic parameters and blood sample collection for humoral parameters. The left femoral artery and vein were cannulated for measurement of hemodynamic data including mean atrial pressure (MAP), and then a balloon‐tipped thermodilution catheter (American Edwards Laboratory, CA) was inserted to measure right atrial pressure (RA), cardiac output (CO), and pulmonary capillary wedge pressure (PCWP). Heart rate (HR) was measured by electrocardiogram based on R‐R interval. Systemic vascular resistance (SVR) was calculated as (MAP‐RAP)/CO. An arterial line was inserted into the femoral artery (for measurement of MAP and blood sampling) and two venous lines were inserted into the femoral vein (for inulin and saline injection). The ureter was cannulated via a left lateral flank for timed urine collection. The study protocol started with a weight adjusted inulin bolus. Inulin was continuously infused at a rate of 1 mL/min for measurement of glomerular filtration rate (GFR). In addition, saline was infused at a rate of 1 mL/min. Urine was collected over a 30 min period and blood samples were collected on ice for volume, electrolytes and assays. Urine collected for cGMP analysis was heated to >90°C before storage. All data collection was performed during continuous 240 bpm pacing. We also initiated the experiment for hemodynamics and sample collections in the normal group. After the experiments, canines were euthanized with an over‐dose of pentobarbital under maintenance anesthesia. The tissue from heart (left atrium (LA) and ventricle (LV)) and kidney (kidney cortex (KC) and medulla (KM)) were harvested. For the further tissue analysis, we carefully harvested tissues from the free wall of LA or LV, and the mid‐section of the KC or KM.

### Assays for circulating neurohumoral factors

Plasma ANP, BNP, cGMP, ANG‐II, aldosterone and plasma renin activity levels were measured by radioimmunoassay as described previously (Ichiki et al. 2013; McKie et al. 2014).

### Histological analysis for structure and fibrosis

Fixed canine tissues were dehydrated, embedded in paraffin, and sectioned at a thickness of 4 *μ*m. Tissue sections were stained with hematoxylin and eosin (H&E) to assess structural changes. Measurement of Collagen content was performed using picrosirius red staining (Ichiki et al. 2013). An Axioplan II KS 400 microscope (CarlZeiss, Germany) was used to capture at least 5 randomly selected images from each slide using a 20X objective, and KS 400 software was used to determine fibrotic area as a percentage of total tissue area.

### RT‐PCR array for canines

Total RNA was isolated from frozen tissue using the Trizol (Invitrogen, CA) method and prepared RNA was treated with DNase I (Invitrogen, CA) (Ichiki et al. 2013, 2014). Total RNA was then reverse transcribed to synthesize cDNA, after genomic DNA elimination, using the RT^2^ First Strand Kit (QIAGEN, Germany) according to the manufacturer's protocol. Custom 96‐well microarrays for canines (custom designed RT^2^ Profiler PCR array; QIAGEN, Germany) for quantification of 179 genes related to RAAS, the NP system, inflammatory cytokines and growth factors, renal injury, apoptosis and fibrosis were used for PCR reactions (Detailed information is shown in Table S1). Negative and positive controls and GAPDH mRNA as a housekeeping gene were included on each custom plate. cDNA samples were mixed with RT^2^ SYBR Green Mastermix and dispensed into the RT^2^ Profiler PCR Array (Berent‐Maoz et al. 2012). The array plates were cycled and Ct values were analyzed using the Lightcycler 480 (Roche, Switzerland). Each data set was analyzed by the ΔΔCt method and normalized to GAPDH expression (Heinze et al. 2012).

### ELISA for cytokines

Total protein was extracted from canine LA, LV, KC, and KM and the concentration determined by BCA assay. Cytokine expression in tissue for monocyte chemoattractant protein (MCP)‐1, IL‐6, IL‐1 beta, or TNF alpha were analyzed by ELISA (Duoset for canines, R&D systems, MN) according to the manufacturer's protocol. Each sample concentration was normalized to protein concentration. Intra‐ and Inter‐assay variations of canine MCP‐1, IL‐6, IL‐1 beta and TNF alpha were 4.3% and 6.1%, 8.7% and 10.9%, 4.3% and 9.0%, and 2.6% and 5.1%, respectively.

### Statistical analysis

All data were expressed as means ± SEM. Data between two groups were assessed by Non‐parametric analysis, Wisconsin 2‐sample test. Statistical significance was accepted at *P* < 0.05.

## Results

### Characteristics of experimental ES‐HF in canines

Table 1 reports characteristics of normal and ES‐HF canines. Similar to our previous study (Ichiki et al. 2013), ES‐HF canines showed significantly lower EF, higher PAP as well as PCWP, and tended to have lower CO and higher LA weights compared to normals. Urine flow and urinary sodium and potassium excretion were significantly lower, and GFR trended to be reduced. Plasma BNP, ANP, cGMP, ANG II, aldosterone, and renin were all elevated compared to normal (Table 1). Thus, the model has hemodynamic failure due to rapid pacing with impaired renal function and activation of the natriuretic peptide system and RAAS.

**Table 1:** Canine characteristics

	Normal (*n* = 4)	ES‐HF (*n* = 4)
General information		
Body weight (BW), kg	21.0 ± 0.5	24.9 ± 1.2
Heart weight (HW), g	191.9 ± 16.8	176.3 ± 4.8
HW/BW	8.2 ± 0.6	7.10 ± 0.3
Left atrium, g	10.3 ± 1.1	**15.6** ± **1.3** a
Left ventricle, g	129.7 ± 12.9	103.2 ± 1.4
Right kidney, g	59.9 ± 6.0	50.9 ± 4.5
Echo parameter		
Ejection fraction, %	62.3 ± 7.3	**24.3** ± **3.7** a
LVDd, mm	41.7 ± 2.5	44.3 ± 3.8
Hemodynamic parameters		
Heart rate, bpm	108.8 ± 5.9	**240** a
Mean arterial pressure, mmHg	113.3 ± 2.2	**95.5** ± **4.1** a
Pulmonary artery pressure, mmHg	14.3 ± 1.1	**25.7** ± **1.8** a
Right atrium pressure, mmHg	2.8 ± 0.8	6.1 ± 1.5
Pulmonary capillary wedge pressure, mmHg	5.7 ± 0.4	**20.0** ± **1.5** a
Cardiac output, l/min	3.5 ± 0.4	2.3 ± 0.2
Systemic vascular resistance, mmHg/l/min	33.3 ± 4.7	47.2 ± 5.6
Renal functions		
Glomerular filtration rate, mL/min	38.4 ± 2.8	32.2 ± 1.6
Urine flow, mL/min	0.36 ± 0.04	**0.22** ± **0.008** a
Urinary sodium excretion, mEq/min	33.7 ± 3.9	**14.7** ± **1.1** a
Urinary potassium excretion, mEq/min	57.7 ± 4.1	**27.6** ± **3.4** a
Neurohumoral factors		
Plasma A‐type natriuretic peptide, pg/mL	24.9 ± 7.1	**97.4** ± **21.0** a
Plasma B‐type natriuretic peptide, pg/mL	13.6 ± 3.5	**35.2** ± **5.3** a
Plasma cGMP, pmol/mL	6.9 ± 0.9	**22.5** ± **3.7** a
Plasma ANG‐II, pg/mL	10.8 ± 3.1	**31.7** ± **4.1** a
Plasma aldosterone, ng/dL	4.8 ± 0.8	**13.0** ± **2.7** a
Plasma renin activity, pg/mL	1.5 ± 0.4	**10.1** ± **2.1** a

Data are expressed as mean ± SEM.

a *P*<0.05 versus normals.

Bold indicates significant values.

### Cardiorenal structure and fibrosis in ES‐HF

**Figure 1:**
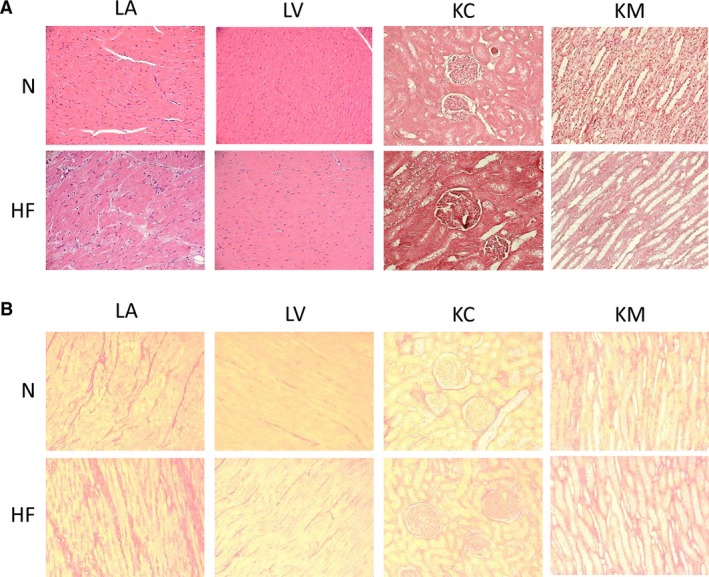
Histological analysis for morphology and fibrosis. (A) Representative H&E stainings. (B) Representative Picrosirius Red staining for fibrosis. Magnification is 20×.

Figure 1A illustrates H&E staining of the heart and kidney. Normal LA and LV showed no abnormalities. Staining of the ES‐HF LA revealed interstitial edema with more interstitial cells, which were not observed in ES‐HF LV (Fig. 1A). Although no remarkable morphological change was observed in normal KC and KM, vacuolization of the distal tubules was observed in ES‐HF KC, consistent with mild renal injury (Fig. 1A).

Picrosirius Red staining showed changes in fibrosis (Fig. 1B). More fibrosis was significantly observed in LA (Normal; 13.2 ± 3.0%, HF; 22.2 ± 1.0%, *P* < 0.05), as has been previously reported (Ichiki et al. 2013) and tended to be higher in KC (Normal; 6.6 ± 1.4%, HF; 9.9 ± 1.3%, p = ns), but not in LV (Normal; 2.8 ± 0.7%, HF; 2.8 ± 0.7%, p = ns) or KM (Normal; 14.0 ± 2.6%, HF; 13.7 ± 1.2%, p = ns).

Taken together, our model had atrial remodeling without ventricular fibrosis as previously reported (Ichiki et al. 2013), and also mild tubular injury.

### Gene expressions of NP system and RAAS in kidney and heart

As reported above, the NP system and RAAS were markedly activated in the ES‐HF circulation. We examined gene expression of both systems in ES‐HF kidney and heart (Table 2). Regarding these expressions in the heart, the patterns of ANP, BNP, CNP and the proNPs processing enzymes corin and furin followed our previously reported pattern in atrium and ventricle (Ichiki et al. 2013); ANP, BNP and furin mRNA expression were higher but corin mRNA tended to be lower in LA. New findings in the heart were related to NP receptor‐B, and degrading enzymes DPP4 and IDE which were higher in LA but not LV. In the kidney, CNP and NPR‐C were up‐regulated representing the first report of activation of these two genes in the kidney in ES‐HF.

**Table 2:** Fold changes of NPs and RAAS gene expressions in ES‐HF kidney and heart

Gene name	Symbol	KC	KM	LA	LV
Natriuretic peptide system					
Atrial natriuretic peptide, ANP	NPPA	1.6	1.4	**3.7** a	1.0
B‐type natriuretic peptide, BNP	NPPB	1.9	1.6	**4.8** a	0.3
C‐type natriuretic peptide, CNP	NPPC	**1.5** a	0.7	2.8	**6.1** a
Natriuretic peptide receptor A	NPR1	1.3	0.6	1.1	0.9
Natriuretic peptide receptor B	NPR2	1.2	0.8	**1.8** a	1.0
Natriuretic peptide receptor C	NPR3	**2.3** a	**2.5** a	1.5	0.9
Corin	CORIN	1.6	0.4	0.8	1.2
Furin	FURIN	1.2	1.0	**3.4** a	**1.8** a
Dipeptidyl peptidase‐4	DPP4	1.4	0.1	**3.0** a	0.8
Insulin degrading enzyme	IDE	1.0	0.9	**2.2** a	1.0
Membrane metalloprotease (neprilysin, NEP)	MME	1.0	0.1	0.0	0.4
Renin‐angiotensin‐aldosterone system					
Angiotensinogen	AGT	1.1	0.5	0.5	**0.1** a
Angiotensin II receptor type I, AT1 receptor	AGTR1	1.3	0.2	1.4	1.6
Renin	REN	**1.7** a	0.1	**12.5** a	1.6
Mineralocorticoid (aldosterone) receptor	NR3C2	0.9	0.6	0.9	0.8

Data are expressed as fold change compared to normals.

a *P*<0.05 versus normals.

Bold indicates significant values.

With regard to RAAS, only renin was significantly up‐regulated and that occurred in the LA and KC. Therefore, the NP system was activated in the LA, however only CNP, NPR‐C and renin were activated in KC in ES‐HF.

### Global gene expression profile of heart and kidney in ES‐HF

**Figure 2:**
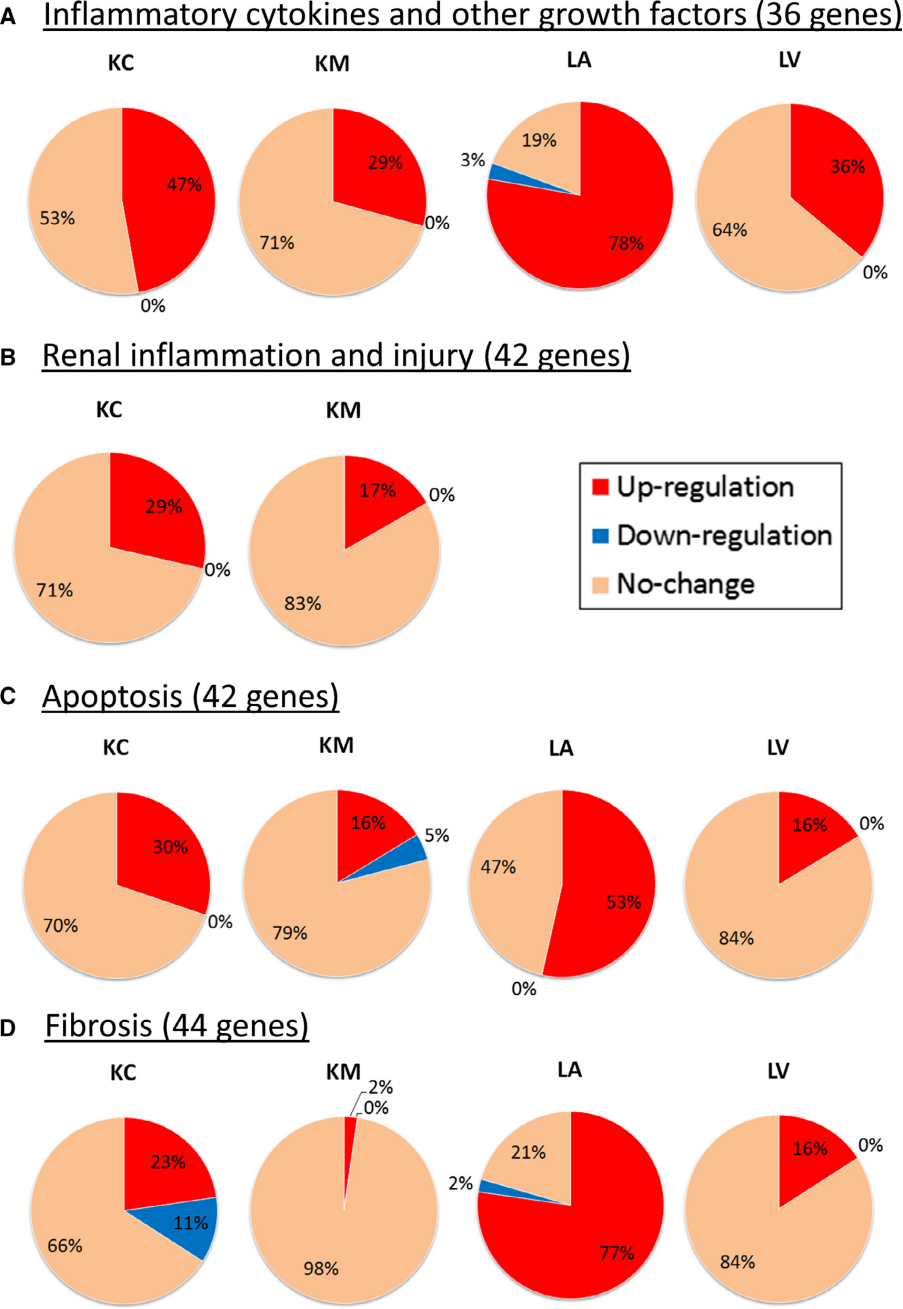
Summary of gene modifications in ES‐HF compared to normal tissues by pathway. Each pie graph illustrates the percentage of up‐regulated genes (red), down‐regulated genes (blue) and un‐changed genes (orange) in each organ for inflammatory cytokines and other growth factors (A), renal inflammation and injury (B), apoptosis (C), and fibrosis (D). Significantly changed gene compared to normal tissues for each organ (p<0.05) were counted.

We examined gene expression profiles related to inflammatory cytokines and growth factors (Fig. 2A), renal inflammation and injury (Fig. 2B), apoptosis (Fig. 2C), and fibrosis (Fig. 2D) in kidney (KC and KM) and heart (LA and LV). All raw data and each fold change compared to normal tissue are shown in Tables S2 and S3.

In Figure 2, genes related to inflammation, renal inflammation and injury, apoptosis and fibrosis were up‐regulated in KC (47%, 29%, 30%, 23%, respectively) and KM (29%, 17%, 16%, and 2%, respectively) although the changes were less than in LA (LA, 78%, 53%, and 77%; and LV, 36%, 16%, and 16%, respectively). Inflammation related genes were mainly up‐regulated in KC, while changes in KM and LV were minor compared to KC and LA.

Genes with the most marked changes (more than fivefold in either the kidney, heart or both compared to normal tissues) are shown in Table 3. As in Figure 2, most of the changes occurred in the KC and LA with less in KM and LV. Several inflammatory and renal injury genes in KC were up‐regulated over 50‐fold, similar to LA (e.g., 139.0‐fold for SELE and 55.9‐fold for ICAM1 in KC and 293.7‐fold for IL6, 143.8‐fold for CCL2 and 133.6‐fold for CSF3, and 86.7‐fold for SERPINE1 in LA), while apoptosis and fibrosis genes were generally up‐regulated less than 50‐fold. Therefore, the kidney displays up‐regulation of genes in the order of inflammation, renal injury, apoptosis and fibrosis in ES‐HF.

**Table 3:** Fold changes from normals (>5‐fold in at least one tissue type examined)

Gene name	Symbol	KC	KM	LA	LV
Inflammatory cytokines and other growth factors					
Tumor necrosis factor‐alpha	TNF	**3.6** a	1.8	**22.0** a	**3.5** a
Interleukin‐6	IL6	**24.7** a	2.5	**293.7** a	**40.0** a
Leukemia inhibitory factor	LIF	**23.0** a	**42.4** a	**45.7** a	**3.8** a
Endothlin‐1	EDN1	3.4	1.4	**9.6** a	1.3
Monocyte chemoattractant protein‐1, MCP‐1	CCL2	**17.0** a	**10.4** a	**143.8** a	**27.7** a
Interleukin‐1 beta	IL1B	**4.4** a	0.5	**10.2** a	4.9
Interleukin‐10	IL10	0.9	0.4	**4.3** a	0.5
Interferon‐gamma	IFNG	**4.1** a	**4.7** a	**21.9** a	**6.3** a
Colony stimulating factor 3	CSF3	**7.4** a	**2.7** a	**133.6** a	**48.8** a
Platelet‐derived growth factor B	PDGFB	**11.7** a	2.4	**2.3** a	0.8
Platelet‐derived growth factor receptor B	PDGFRB	**1.7** a	0.8	**2.9** a	1.3
Vascular cell adhesion molecule‐1	VCAM1	**37.2** a	**4.3** a	**33.7** a	**10.4** a
C‐reactive protein	CRP	**7.1** a	1.3	0.0	3.2
Insulin	INS	1.3	1.0	**7.0** a	2.8
Von Willebrand factor	VWF	1.0	0.6	**4.3** a	1.3
Tissue plasminogen activator	PLAT	**3.9** a	**1.8** a	**13.5** a	**3.1** a
Plasminogen activator inhibitor‐1	SERPINE1	**26.5** a	**12.4** a	**86.7** a	**28.9** a
Nuclear factor kappa‐B	NFKB1	**5.5** a	**2.5** a	**7.2** a	**4.0** a
Renal inflammation and injury					
Interleukin‐1 alpha	IL1A	**7.2** a	**3.4** a	–	–
Interleukin‐8	IL8	**38.4** a	**15.6** a	–	–
Granulocyte‐colony stimulating factor	CSF3	**8.5** a	3.0	–	–
Intercellular adhesion molecule 1	ICAM1	**55.9** a	**14.7** a	–	–
E‐selectin	SELE	**139.0** a	**53.6** a	–	–
Serum amyloid A1	SAA1	**13.8** a	1.5	–	–
Immediate early response 3	IER3	**8.6** a	**3.0** a	–	–
Apoptosis					
Baculoviral IAP repeat containing 3	BIRC3	**25.9** a	**14.9** a	**25.2** a	**15.9** a
Fas (TNF superfamily member 6)	FAS	**4.2** a	1.7	**8.1** a	**2.8** a
TNF receptor superfamily member 12A	TNFRSF12A	**4.6** a	**7.1** a	**11.0** a	1.5
Myc	MYC	**7.7** a	**3.4** a	**4.7** a	**3.3** a
Fibrosis					
Collagen type I	COL1A1	**1.8** a	0.4	**60.6** a	2.2
Collagen type III	COL3A1	1.0	0.5	**16.0** a	1.6
Collagen type IV	COL4A1	**1.7** a	1.1	**9.3** a	20.9
Collagen type IV	COL4A2	**1.5** a	0.9	**8.9** a	1.8
Fibronectin‐1	FN1	**1.9** a	1.0	**24.2** a	2.0
Connective tissue growth factor	CNGF	**1.9** a	0.9	**6.0** a	**1.5** a
General transcription factor IIIA	GTF3A	**0.1** a	0.4	1.2	0.8
Integrin beta‐3	ITGB3	**2.8** a	0.8	**10.1** a	**4.1** a
Matrix metalloproteinase 8	MMP8	**16.5** a	3.6	4.1	6.4
Matrix metalloproteinase 9 (gelatinase B)	MMP9	**0.3** a	3.0	**5.7** a	4.8
Matrix metalloproteinase 13	MMP13	**20.6** a	3.6	**10.6** a	1.9
Tissue inhibitor of metalloproteinases 1	TIMP1	**0.3** a	1.2	**15.9** a	2.5
Transforming growth factor‐beta 2	TGFB2	**1.2** a	1.2	**6.6** a	**1.6** a
Transforming growth factor‐beta 3	TGFB3	2.2	0.9	**10.2** a	0.9
Transforming growth factor‐beta receptor 1	TGFBR1	**0.2** a	1.2	**6.3** a	**2.0** a
Latent‐transforming growth factor beta binding protein‐1	LTBP1	1.5	1.0	**6.5** a	1.5

Data are expressed as fold change compared to normals.

a *P*<0.05 versus normals.

Bold indicates significant values.

### Protein expression of inflammatory cytokines in kidney of ES‐HF

**Figure 3:**
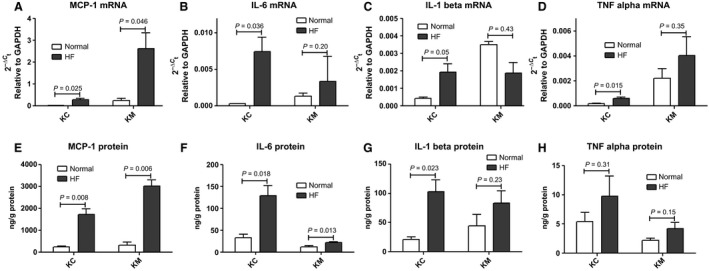
Expression of inflammatory cytokines. mRNA expression of MCP‐1 (A), IL‐6 (B), IL‐1 beta (C), and TNF alpha (D) were measured by PCR array. Protein expression of MCP‐1 (E), IL‐6 (F), IL‐1 beta (G), and TNF alpha (H) were measured by ELISA. Data are expressed as mean ± SEM.

Next, we investigated highly modified inflammatory genes in the kidney to define gene changes at the protein expression level. For inflammatory cytokines, we examined MCP‐1, IL‐6, IL‐1 beta and TNF alpha which were up‐regulated in kidney (Fig. 3A–D). MCP‐1 (Fig. 3E) and IL‐6 (Fig. 3F) protein were up‐regulated in both KC and KM. IL‐1 beta protein (Fig. 3G) was up‐regulated significantly in KC, following the same pattern as the gene changes. TNF alpha proteins tended to be higher in KC and KM, but not significantly (Fig. 3H).

In summary, inflammatory changes were observed at both the gene and protein levels in kidney, but mainly in the KC.

## Discussion

The current study was designed to advance our understanding of the kidney in ES‐HF with a special focus on the NP system, RAAS, and deleterious molecular gene pathways. The major findings of our study are that early evolving experimental HF produced by rapid ventricular pacing results in mild tubular injury in the kidney cortex, and widespread gene pathway activation, not only of the NP system in the atrium, but also inflammation, renal injury, apoptosis and fibrosis pathways with activation of renin, CNP, and NPR‐C in the kidney. These findings may have significant implications for HF therapies identifying renal targets for novel therapeutics in the early phase of HF.

As expected, our canine HF model was characterized by reduced EF and blood pressure, elevated PCWP and PAP and marked activation of the circulating NPs and RAAS along with sodium and water retention (Ichiki et al. 2013). In terms of remodeling, our canine ES‐HF had only atrial remodeling, not ventricular fibrosis. Thus, this model of ES‐HF mimics key features of human ES‐HF with cardiorenal impairment and neurohumoral activation. A hallmark of HF is a progressive decline in renal function which has been termed cardiorenal syndrome. Indeed, renal impairment in HF and/or acute kidney injury, is now recognized as a powerful independent predictor of outcomes (Schefold et al. 2016). Despite such a key role for the kidney in HF, there remains a lack of insight and information on the renal cellular response to HF which may provide insights into potential progressive renal dysfunction as well as structural remodeling of the kidney. Although the use of novel renal biomarkers have recently been useful in diagnosis and therapy of the cardiorenal syndrome, little is known about the earliest effects of HF on the kidney (Brisco and Testani 2014).

As expected, we observed atrial fibrosis (Haneda et al. 1991). Indeed, Hanna and colleagues also reported atrial fibrosis in a canine model of HF with substantially more fibrosis present in the LA than LV (Hanna et al. 2004). In addition, this group showed an increase in tissue apoptosis, inflammatory cell infiltration, and cell death that was greater in the LA than the LV. Here we extend these observations by investigating a much broader panel of genes in these pathways. It is well known that gene and protein activation occur in the order of inflammation, apoptosis and fibrosis in the HF heart (Braunwald 2013), therefore combined, these studies suggest that the LA becomes impaired first in ES‐HF beginning with inflammatory pathway activation followed by apoptosis and fibrosis while the LV lags behind in damage with inflammatory responses beginning, but little apoptosis or fibrosis. Notably, this study provides a first attempt to explore gene patterns in both the kidney and heart, focusing on a wide range of inflammatory and renal injury genes in response to experimental HF in our canine model. A limitation however is that in this first attempt to understand a multi‐organ response, we examined changes at only one time point. Therefore, further studies are needed to explore how these gene responses are changed over the entire spectrum HF development.

As we observed the greatest gene changes as well as fibrosis in the LA in the current study, we speculate that factors may be released from the LA into the circulation that could affect the KC early, particularly as the function of the KC is to filter plasma fluids from the circulation coming from the LA. Indeed, as Virzi et al. (2015) have reported, plasma from patients with acute HF possesses factors that, in cultured renal tubular cells, mediate renal tubular injury and apoptosis. More recently, studies have reported the release of microRNA's related to cardiac fibrosis was found in plasma of human HF (Li et al. 2016). Our findings are consistent with these reports, as well as with the fact that inflammatory and fibrotic pathways are known contributors of end‐stage kidney disease (Chatziantoniou et al. 2004), implicating them as potential early treatment target pathways in HF. Further, as the kidney is the site of initial renin activation, specifically localized to the KC, there may be a fundamental mechanism of cardiorenal injury through increased circulatory levels of RAAS and modulation of renal gene expression which would be an important therapeutic target for renal protection. Hemodynamic failure with lower perfusion pressure in the kidney also plays a role in KC injury in HF.

Employing a strategy which involved determining gene expression of 179 selected genes, we examined the KC and KM for changes in inflammatory, renal injury, apoptotic, and fibrotic genes after inducing ES‐HF. The major finding of our study was the demonstration of gene activation in both the KC and KM involving these key pathways. We also validated 4 genes from the inflammatory pathways at the protein levels; MCP‐1, IL‐6, IL1 beta and TNF alpha. All showed activation at the protein level although the increases in TNF alpha strongly trended toward an increase but did not achieve significance. These activated pathways may play an important role in the mild renal injury detected by H&E staining in the KC (Fig. 1A). The KM showed fewer gene changes compared to KC, particularly of inflammatory cytokines at the gene and protein levels. These data suggest that the KC becomes injured in ES‐HF prior to KM damage with the same pathways activated sequentially as in the heart, which is in contrast with the prevailing theory of medullary ischemia as a driver in HF (Goldfarb et al. 2001). Since there are few studies demonstrating concurrent tissue injury of the medulla and cortex, our finding is very unique and establishes cortical injury which is more prominent than medullary changes in ES‐HF. Regarding mechanism, it is widely thought that only systemic RAAS activation mediates organ injury, however we speculate that in the kidney, intrarenal RAAS plays an important role in renal injury and disease progression, particularly in the cortex (Yang and Xu 2017).

The activation of RAAS may counteract protective properties through stimulation of phosphodiesterase or impairment in NP receptor function while NPs may mediate protective actions in multiple cell types of the heart and kidney (Haneda et al. 1991; Volpe et al. 2014), suggesting members of these systems may be important therapeutic targets. Autonomic nervous system activity may also play an important role in the link between the heart and kidney in this model. Piccirillo et al. found that sympathetic nerve activity assessed by QT‐interval was augmented in HF (Piccirillo et al. 2009), and Ng et al. (2011) reported that autonomic and electrophysiological remodeling occurs in the LA in a chronic pacing model of HF in canines. Over time, circulating NPs, RAAS, and inflammatory markers increased which were attenuated by chronic vagus nerve stimulation or renal sympathetic denervation (Zhang et al. 2009; Zhao et al. 2014), underscoring an important therapeutic target could involve the autonomic nervous system. Of note, canines were paced much longer in these studies than in our model of ES‐HF, suggesting these pathways may be activated later in HF, however further studies are needed for comparison.

The myocardial NP expression was activated as expected, however we importantly report for the first time, the increase of CNP mRNA in the KC in ES‐HF. Studies support a role for renal CNP as a regulator of cell proliferation and endothelial repair and regeneration whose gene expression is increased by hypoxia, cytokines and fibrotic growth factors (Doi et al. 2001; Sangaralingham et al. 2011; Ichiki et al. 2014). The current studies support further studies of renal CNP as a potential novel therapeutic for renal protection in HF.

A limitation to our study is the relatively small number of canines in each group. However, this large animal model of ES‐HF compared to small animal models lacks hemodynamic variability and is well established as a model of dilated cardiomyopathy in studies from our group as well as other groups (Spinale et al. 1997; Marin‐Garcia et al. 2001; Trochu et al. 2003), providing statistical significance with smaller numbers of animals. Any animal model of HF has limitations as a key AHA statement summarized which stated that “there are shortcomings to all HF animal models that limit their relevance to disease in human” (Houser et al. 2012). Nonetheless, we think that using large animal models gives very important information including hemodynamic and cardiac remodeling information mimic human HF much more closely than small animal models. As a model of dilated cardiomyopathy, we need to keep in mind that the changes may not be applicable in other HF states, such as ischemic cardiomyopathy. A second limitation which should be addressed in future studies, is the temporal evolution of these changes overtime.

### Perspectives and significance

Our findings have clinical implications. Specifically, they lay the foundation for further studies of potential pharmacological interventions targeting these multiple molecular pathways in the kidney which could contribute to progressive renal impairment in ES‐HF. The NPs are activated both in the heart and kidneys in our model, along with activation of RAAS, supporting studies where pharmacological interventions such as ACE inhibitor/NEP inhibitor, which co‐target the RAAS and NPs, have promising reno‐cardiac protection in HF (Packer et al. 2015).

## Conclusions

In conclusion, the present study has demonstrated the adaption of the kidney and heart to the ES‐HF in a large animal model with a focus on gene pathways involving inflammatory cytokines as well as genes of renal injury, apoptosis, and fibrosis. Changes in these gene pathways were associated with evidence for mild renal injury in the renal cortex. Thus, our findings may provide important clues into cardiorenal pathophysiology and guide the use of the novel therapeutics in ES‐HF for cardiorenal protection.

## Conflict of Interest

None.

## Data Accessibility

## Supporting information




**Table S1:** Description of genes in RT2‐PCR array.
**Table S2:** Gene expression levels in normals vs HF.
**Table S3:** Fold changes of gene expressions from normals.
