# Supplementary material for: Early activation of deleterious molecular pathways in the kidney in experimental heart failure with atrial remodeling
Source: Physiol Rep. 2017 May 15;5(9):e13283. doi: 10.14814/phy2.13283 (PMC5430128; doi:10.14814/phy2.13283)
Supplement: Supplementary file 1 — Table S1: Description of genes in RT2‐PCR array. Table S2: Gene expression levels in normals vs HF. Table S3: Fold changes of gene expressions from normals. [file PHY2-5-e13283-s001.docx]

**Supplemental Table 1. Description of genes in RT^2^-PCR array**

| **Gene Name** | **Symbol** | **Unigene** | **GenBank** |
| --- | --- | --- | --- |
| ***Renin-Angiotensin-Aldosterone System (4 genes)*** |  |  |  |
| Angiotensinogen | AGT | Cfa.3646 | XM_536348 |
| Angiotensin II receptor type I, AT1 receptor | AGTR1 | Cfa.3699 | XM_542827 |
| Renin | REN | Cfa.3701 | NM_001003194 |
| Mineralocorticoid (Aldosterone) Receptor | NR3C2 | Cfa.32999 | XM_532685 |
| ***Natriuretic Peptide System (11 genes)*** |  |  |  |
| Atrial natriuretic peptide, ANP | NPPA | Cfa.1452 | XM_845264 |
| B-type natriuretic peptide, BNP | NPPB | N/A | XM_544566 |
| C-type natriuretic peptide, CNP | NPPC | N/A | XM_847591 |
| Natriuretic peptide receptor A | NPR1 | Cfa.23540 | XM_547577 |
| Natriuretic peptide receptor B | NPR2 | Cfa.12543 | XM_531993 |
| Natriuretic peptide receptor C | NPR3 | N/A | XM_850349 |
| Corin, serine protease | CORIN | Cfa.670 | XM_539254 |
| Furin, paired basic amino acid cleaving enzyme | FURIN | Cfa.21237 | XM_545864 |
| Dipeptidyl Peptidase-4 | DPP4 | Cfa.15116 | XM_535933 |
| Insulin degrading enzyme | IDE | Cfa.47318 | XM_534963 |
| Membrane metalloprotease (neprilysin, NEP) | MME | Cfa.13894 | XM_534313 |
| ***Inflammatory cytokines and other growth factors (36 genes)*** |  |  |  |
| Tumor Necrosis Factor-alpha | TNF | Cfa.54 | NM_001003244 |
| Interleukin-6 | IL6 | Cfa.3528 | NM_001003301 |
| Glycoprotein 130 | IL6ST | Cfa.33585 | XM_535239 |
| Leukemia inhibitory factor | LIF | Cfa.150 | XM_534732 |
| Endothlin-1 | EDN1 | Cfa.125 | NM_001002956 |
| Monocyte chemoattractant protein-1, MCP-1 | CCL2 | Cfa.3851 | NM_001003297 |
| Chemokine receptor 2 | CCR2 | N/A | XM_541906 |
| Interleukin-1 beta | IL1B | Cfa.33592 | NM_001037971 |
| Interleukin-10 | IL10 | Cfa.38 | NM_001003077 |
| Interleukin-13 | IL13 | Cfa.3566 | NM_001003384 |
| Interferon-gamma | IFNG | Cfa.3900 | NM_001003174 |
| Nerve growth factor receptor | NGFR | N/A | XM_548191 |
| Colony stimulating factor 3 | CSF3 | N/A | XM_845213 |
| Hepatocyte growth factor | HGF | Cfa.3443 | NM_001002964 |
| Epidermal growth factor | EGF | Cfa.3524 | NM_001003094 |
| Platelet-Derived Growth Factor A | PDGFA | Cfa.35680 | NM_001190172 |
| Platelet-Derived Growth Factor B | PDGFB | Cfa.3913 | NM_001003383 |
| Platelet-Derived Growth Factor Receptor B | PDGFRB | Cfa.3909 | NM_001003382 |
| Vascular endothelial growth factor A | VEGFA | Cfa.3581 | NM_001110502 |
| Vascular cell adhesion molecule-1 | VCAM1 | Cfa.1941 | NM_001003298 |
| Ras homolog gene family member A | RHOA | Cfa.20231 | NM_001003273 |
| Rho-associated coiled-coil containing protein-1 | ROCK1 | Cfa.3381 | XM_537305 |
| Rho-associated coiled-coil containing protein-2 | ROCK2 | Cfa.40416 | XM_540083 |
| C-reactive protein | CRP | N/A | XM_545746 |
| Insulin | INS | Cfa.18796 | NM_001130093 |
| Von Willebrand factor | VWF | Cfa.111 | NM_001002932 |
| Tissue plasminogen activator | PLAT | N/A | XM_539955 |
| Plasminogen | PLG | Cfa.3464 | XM_533468 |
| Plasminogen activator inhibitor-1 | SERPINE1 | Cfa.109 | XM_853392 |
| Adiponectin | ADIPOQ | Cfa.3487 | NM_001006644 |
| Mitogen-activated protein kinase 1 | MAPK1 | Cfa.2796 | NM_001110800 |
| RAC-alpha serine/threonine-protein kinase 1 | AKT1 | N/A | XM_548000 |
| Signal transducer and activator of transcription 1 | STAT1 | Cfa.720 | XM_545571 |
| Signal transducer and activator of transcription 3 | STAT3 | Cfa.18421 | XM_548090 |
| Nuclear factor kappa-B | NFKB1 | Cfa.10766 | NM_001003344 |
| Transcription factor Sp1 | SP1 | Cfa.7309 | XM_543633 |
| ***Renal inflammation and injury (42 genes)*** |  |  |  |
| Interleukin-1 alpha | IL1A | Cfa.3645 | NM_001003157 |
| Interleukin-7 | IL7 | Cfa.36752 | NM_001048138 |
| Interleukin-8 | IL8 | Cfa.3510 | NM_001003200 |
| Interleukin-18 | IL18 | Cfa.40 | NM_001003169 |
| Granulocyte Macrophage colony-stimulating Factor | CSF2 | Cfa.3784 | NM_001003245 |
| granulocyte-colony stimulating factor | CSF3 | N/A | XM_845213 |
| Insulin-like growth factor 1 | IGF1 | Cfa.3888 | XM_848024 |
| Neural cell adhesion molecule 1 | NCAM1 | Cfa.11012 | NM_001010950 |
| Intercellular Adhesion Molecule 1 | ICAM1 | Cfa.3842 | XM_542075 |
| P-selectin | SELP | Cfa.3842 | XM_537202 |
| E-selectin | SELE | Cfa.3868 | NM_001003310 |
| Chemokine (C-C motif) ligand 5, RANTES | CCL5 | Cfa.21 | NM_001003010 |
| C-C chemokine receptor type 1 | CCR1 | Cfa.24665 | NM_001038606 |
| C-C chemokine receptor type 3 | CCR3 | Cfa.30412 | NM_00105261 |
| Serum amyloid A1 | SAA1 | Cfa.27722 | NM_001003050 |
| N-acetyl-beta-(D)-glucosaminidase, NAG | MGEA5 | Cfa.12884 | XM_534996 |
| kidney injury molecule-1, KIM-1 hepatitis A virus cellular receptor1 | HAVCR1 | N/A | XM_849795 |
| Annexin A5 | ANXA5 | Cfa.12149 | XM_533303 |
| Thrombospondin 1, TSP-1 | THBS1 | Cfa.21204 | XM_544610 |
| Calponin 1 | CNN1 | Cfa.41686 | XM_542053 |
| Fibulin-1 | FBLN1 | Cfa.45531 | XM_531698 |
| Ankyrin1 | ANK1 | Cfa.16256 | XM_539957 |
| Spectrin1 | SPTA1 | N/A | XM_545733 |
| Heat shock protein 27 | HSPB1 | Cfa.3849 | NM_001003295 |
| Toll-like receptor 2 | TLR2 | Cfa.15802 | NM_001005264 |
| S100 calcium-binding protein A4 | S100A4 | Cfa.3649 | NM_001003161 |
| S100 calcium-binding protein A6 | S100A6 | Cfa.4306 | XM_537268 |
| Clusterin, apolipoprotein J | CLU | Cfa.1254 | NM_001003370 |
| Glutathione S-transferase alpha3 | GSTA3 | Cfa.40762 | XM_532173 |
| neutrophil gelatinase-associated lipoprotein  (NGAL), Lipocalin 2 (LCN2) | LCN2 | Cfa.38380 | XM_548441 |
| Trefoil factor 3 | TFF3 | Cfa.40282 | NM_001002990 |
| FoxP3, forkhead box P3 | FOXP3 | Cfa.47639 | NM_001168461 |
| Immediate early response 3 | IER3 | Cfa.16863 | XM_538829 |
| Signal transducer and activator of transcription 2 | STAT2 | Cfa.21211 | XM_538232 |
| Matrix Gla protein | MGP | Cfa.40454 | XM_848662 |
| Superoxide dismutase 1 | SOD1 | Cfa.6360 | NM_001003035 |
| Catalase | CAT | Cfa.188 | NM_001002984 |
| Na, K-ATPase1 | ATP1A1 | Cfa.3039 | NM_001003306 |
| Na, K-ATPase2 | ATP1A2 | Cfa.37945 | XM_545753 |
| Lysyl oxidase | LOX | N/A | XM_538599 |
| Kruppel-like transcription factor, Zf9 | KLF6 | Cfa.36845 | XM_544279 |
| C4 component | C4A | Cfa.21548 | XM_538843 |
| ***Apoptosis (42 genes)*** |  |  |  |
| Cytochrome c-1 | CYC1 | Cfa.1327 | XM_532351 |
| Apoptotic protease activating factor 1 | APAF1 | N/A | XM_539737 |
| B-cell lymphoma 2 | BCL2 | Cfa.110 | NM_001002949 |
| B-cell lymphoma 2 associated X protein | BAX | Cfa.22 | NM_001003011 |
| Bcl-2 associated death promoter | BAD | Cfa.38968 | NM_001031820 |
| BCL2-antagonist/killer 1 | BAK1 | Cfa.23307 | NM_001020808 |
| BH3 interacting-domain death agonist | BID | N/A | XM_543733 |
| Baculoviral IAP repeat containing 2 | BIRC2 | Cfa.828 | NM_001048023 |
| Baculoviral IAP repeat containing 3 | BIRC3 | Cfa.18376 | NM_001080725 |
| Caspase 2 | CASP2 | Cfa.12400 | NM_001195151 |
| Caspase 3 | CASP3 | Cfa.84 | NM_001003042 |
| Caspase 7 | CASP7 | N/A | XM_544026 |
| Caspase 8 | CASP8 | Cfa.24658 | NM_001048029 |
| Caspase 9 | CASP9 | Cfa.20874 | NM_001031633 |
| CASP and FADD like apoptosis regulator | CFLAR | Cfa.18114 | XM_545592 |
| Conserved helix-loop-helix ubiquitous kinase | CHUK | Cfa.39280 | XM_534990 |
| Death-associated protein 6 | DAXX | Cfa.45731 | XM_858216 |
| Diablo homolog, mitochondrial | DIABLO | Cfa.11132 | NM_001079767 |
| Calcineruin A alpha | PPP3CA | Cfa.3430 | XM_535672 |
| Fas (TNF superfamily member 6) | FAS | Cfa.18344 | XM_543595 |
| Fas Ligand | FASLG | Cfa.4547 | XM_848916 |
| TNF receptor superfamily member 12A | TNFRSF12A | N/A | NM_001193299 |
| TNF receptor superfamily member 1A | TNFRSF1A | Cfa.3498 | XM_849381 |
| TNF receptor superfamily member 21 | TNFRSF21 | Cfa.43060 | XM_847321 |
| TNF receptor superfamily member 25 | TNFRSF25 | N/A | XM_546752 |
| TNF superfamily member 10 | TNFSF10 | Cfa.20893 | NM_001130836 |
| lymphotoxin alpha (TNF superfamily member1) | LTA | N/A | XM_843793 |
| Inhibitor of kappa light polypeptide B gene enhancer in B-cells, kinase beta | IKBKB | Cfa.46114 | XM_539954 |
| Inhibitor of kappa light polypeptide G gene enhancer in B-cells, kinase gamma | IKBKG | N/A | XM_003640238 |
| Apoptosis signal-regulating kinase-1  (ASK1=MAP3K5) | MAP3K5 | Cfa.35678 | XM_533420 |
| Mitogen-activated protein kinase 8 | MAPK8 | Cfa.2669 | XM_534943 |
| Mitogen-activated protein kinase 9 | MAPK9 | Cfa.14141 | XM_851205 |
| Nucleotide-binding oligomerization domain  containing protein 1 | NOD1 | N/A | XM_539499 |
| Myc | MYC | Cfa.3786 | NM_001003246 |
| Nerve growth factor | NGF | Cfa.3899 | NM_001194950 |
| Nerve growth factor receptor | NGFR | N/A | XM_548191 |
| Poly (ADF-ribose) polymerase 1 | PARP1 | Cfa.21541 | XM_547506 |
| Transcription factor p65 | RELA | Cfa.41791 | XM_540850 |
| Calpain-2 | CAPN2 | Cfa.1369 | XM_537240 |
| X-linked inhibitor of apoptosis | XIAP | Cfa.4543 | XM_538165 |
| Tumor protein p53 | TP53 | Cfa.3658 | NM_001003210 |
| Mitochondrial carnitine/acylcarnitine  carrier protein | SLC25A29 | N/A | XM_547978 |
| ***Fibrosis (44 genes)*** |  |  |  |
| Collagen type I | COL1A1 | Cfa.100 | NM_001003090 |
| Collagen type III | COL3A1 | Cfa.3093 | XM_535997 |
| Collagen type IV | COL4A1 | Cfa.208 | XM_534182 |
| Collagen type IV | COL4A2 | Cfa.1455 | XM_534183 |
| Collagen type XV | COL15A1 | Cfa.37504 | XM_532009 |
| Contactin-1 | CNTN1 | Cfa.19192 | XM_534836 |
| Fibronectin-1 | FN1 | Cfa.3707 | XM_536059 |
| Fibroblast growth factor 23 | FGF23 | N/A | XM_849487 |
| Connective Tissue Growth Factor | CNGF | Cfa.1225 | XM_533406 |
| General transcription factor IIIA | GTF3A | Cfa.39671 | XM_534523 |
| Integrin alpha-1 | ITGA1 | N/A | XM_546328 |
| Integrin alpha-2 | ITGA2 | N/A | XM_546326 |
| Growth differentiation factor 7 | GDF7 | N/A | XM_540103 |
| Integrin alpha-chain V | ITGAV | Cfa.45032 | XM_545559 |
| Integrin beta-1 | ITGB1 | Cfa.15441 | XM_535143 |
| Integrin beta-3 | ITGB3 | Cfa.3641 | NM_001003162 |
| Integrin beta-5 | ITGB5 | Cfa.18366 | XM_846152 |
| Decorin (proteoglycan) | DCN | Cfa.3762 | NM_001003228 |
| Beta cytoskeletal action | ACTB | N/A | NM_001195845 |
| Matrix metalloproteinase 1 (Collagenase 1) | MMP1 | N/A | XM_546546 |
| Matrix metalloproteinase 2 (Gelatinase A) | MMP2 | Cfa.3597 | XM_535300 |
| Matrix metalloproteinase 3 | MMP3 | Cfa.3447 | NM_001002967 |
| Matrix metalloproteinase 8 | MMP8 | N/A | XM_546547 |
| Matrix metalloproteinase 9 (Gelatinase B) | MMP9 | Cfa.3470 | NM_001003219 |
| Matrix metalloproteinase 13 | MMP13 | Cfa.3628 | XM_536598 |
| Tissue inhibitor of metalloproteinases 1 | TIMP1 | Cfa.3680 | NM_001003182 |
| Tissue inhibitor of metalloproteinases 2 | TIMP2 | Cfa.3497 | NM_001003082 |
| Tissue inhibitor of metalloproteinases 3 | TIMP3 | Cfa.45034 | XM_538410 |
| Transforming growth factor-beta 1 | TGFB1 | Cfa.3509 | NM_001003309 |
| Transforming growth factor-beta 2 | TGFB2 | Cfa.39067 | XM_545713 |
| Transforming growth factor-beta 3 | TGFB3 | Cfa.24656 | XM_547918 |
| Transforming growth factor-beta receptor 1 | TGFBR1 | N/A | XM_538750 |
| Transforming growth factor-beta receptor 2 | TGFBR2 | Cfa.128 | XM_534237 |
| Transforming growth factor-beta receptor 3 | TGFBR3 | Cfa.37388 | XM_547284 |
| Latent-Transforming growth factor beta binding protein-1 | LTBP1 | Cfa.23392 | XM_857825 |
| SMAD family member 1 | SMAD1 | Cfa.20102 | XM_862216 |
| SMAD family member 2 | SMAD2 | Cfa.25907 | XM_537269 |
| SMAD family member 3 | SMAD3 | Cfa.43487 | NM_001170829 |
| SMAD family member 4 | SMAD4 | Cfa.1648 | XM_853717 |
| SMAD family member 6 | SMAD6 | N/A | XM_544737 |
| SMAD family member 7 | SMAD7 | N/A | XM_547692 |
| Bone morphogenetic protein 2 | BMP2 | Cfa.7153 | XM_854757 |
| Bone morphogenetic protein 4 | BMP4 | N/A | XM_547817 |
| Bone morphogenetic protein 7 | BMP7 | Cfa.8 | XM_534462 |

**Supplemental Table 2. Gene expression levels in normals vs HF.**

|  |  | **KC** | | **KM** | | **LA** | | **LV** | |
| --- | --- | --- | --- | --- | --- | --- | --- | --- | --- |
| **Gene Name** | **Symbol** | **N** | **HF** | **N** | **HF** | **N** | **HF** | **N** | **HF** |
| ***Renin-Angiotensin-Aldosterone System*** |  |  |  |  |  |  |  |  |  |
| Angiotensinogen | AGT | 8.1e-2 ± 1.4e-2 | 9.2e-2 ± 1.3e-2 | 2.7e-3 ± 1.9e-3 | 1.4e-3 ± 1.2e-3 | 3.9e-4 ± 1.7e-4 | 2.0e-4 ± 1.1e-4 | 4.8e-5 ± 3.9e-5 | **4.7e-6 ± 7.0e-7*** |
| Angiotensin II receptor type I, AT1 receptor | AGTR1 | 6.4e-3 ± 8.4e-4 | 8.1e-3 ± 1.0e-3 | 1.2e-2 ± 4.3e-3 | 2.8e-3 ± 1.1e-3 | 7.9e-4 ± 1.5e-4 | 1.1e-3 ± 1.0e-4 | 4.6e-4 ± 4.8e-5 | 7.6e-4 ± 1.6e-4 |
| Renin | REN | 5.5e-3 ± 4.9e-4 | **9.3e-3 ± 2.9e-4*** | 1.8e-3 ± 1.2e-3 | 2.6e-4 ± 5.6e-5 | 3.1e-6 ± 8.9e-7 | **3.9e-5 ± 8.7e-6*** | 1.9e-6 ± 6.9e-7 | 3.0e-6 ± 9.1e-7 |
| Mineralocorticoid (Aldosterone) Receptor | NR3C2 | 1.1e-2 ± 1.8e-3 | 9.7e-3 ± 1.1e-3 | 2.9e-2 ± 4.8e-3 | 1.6e-2 ± 4.0e-3 | 2.6e-3 ± 3.0e-4 | 2.2e-3 ± 2.2e-4 | 1.6e-3 ± 8.8e-5 | 1.4e-3 ± 1.3e-4 |
| ***Natriuretic Peptide System*** |  |  |  |  |  |  |  |  |  |
| Atrial natriuretic peptide, ANP | NPPA | 0.7e-4 ± 5.3e-6 | 1.1e-4 ± 1.2e-5 | 8.6e-5 ± 2.0e-5 | 1.2e-4 ± 5.5e-6 | 0.32 ± 9.6e-2 | **1.21 ± 0.19*** | 2.9e-5 ± 1.5e-5 | 2.8e-5 ± 1.5e-5 |
| B-type natriuretic peptide, BNP | NPPB | 9.8e-6 ± 1.6e-6 | 1.9e-5 ± 4.2e-6 | 9.1e-5 ± 3.7e-5 | 1.5e-4 ± 3.8e-5 | 1.9e-4 ± 6.3e-5 | **9.0e-4 ± 2.8e-4*** | 6.9e-6 ± 1.3e-6 | 2.3e-6 ± 1.2e-6 |
| C-type natriuretic peptide, CNP | NPPC | 1.6e-4 ± 1.3e-5 | **2.4e-4 ± 1.3e-5*** | 1.2e-3 ± 7.6e-4 | 8.1e-4 ± 3.2e-4 | 7.1e-5 ± 1.7e-5 | 2.0e-4 ± 5.8e-5 | 1.7e-5 ± 3.6e-6 | **1.0e-4 ± 7.1e-5*** |
| Natriuretic peptide receptor A | NPR1 | 6.6e-3 ± 7.7e-4 | 8.7e-3 ± 9.3e-4 | 3.5e-2 ± 5.9e-3 | 2.1e-2 ± 3.2e-3 | 2.1e-3 ± 1.8e-4 | 2.5e-3 ± 3.2e-4 | 2.0e-3 ± 1.0e-4 | 1.8e-3 ± 2.9e-4 |
| Natriuretic peptide receptor B | NPR2 | 3.9e-3 ± 3.6e-4 | 4.9e-3 ± 2.5e-4 | 1.1e-2 ± 7.0e-4 | 8.8e-3 ± 1.1e-3 | 2.3e-3 ± 2.0e-4 | **4.2e-3 ± 3.4e-4*** | 1.3e-3 ± 9.3e-5 | 1.4e-3 ± 1.8e-4 |
| Natriuretic peptide receptor C | NPR3 | 3.2e-3 ± 3.0e-4 | **7.2e-3 ± 7.5e-4*** | 1.4e-2 ± 1.5e-3 | **3.4e-2 ± 4.2e-3*** | 1.7e-2 ± 3.3e-3 | 2.6e-2 ± 2.3e-3 | 6.1e-3 ± 2.5e-4 | 5.3e-3 ± 9.4e-4 |
| Corin | CORIN | 1.6e-5 ± 2.4e-6 | 2.6e-5 ± 3.0e-6 | 3.9e-4 ± 1.1e-4 | 1.5e-4 ± 5.9e-5 | 1.3e-2 ± 2.8e-3 | 9.9e-3 ± 1.5e-3 | 4.9e-3 ± 8.8e-4 | 6.1e-3 ± 3.8e-4 |
| Furin | FURIN | 1.2e-2 ± 9.3e-4 | 1.4e-2 ± 1.1e-3 | 3.6e-2 ± 2.4e-3 | 3.4e-2 ± 6.1e-3 | 3.5e-3 ± 3.7e-4 | **1.2e-2 ± 1.8e-3*** | 1.7e-3 ± 1.5e-4 | **3.1e-3 ± 3.9e-4*** |
| Dipeptidyl Peptidase-4 | DPP4 | 0.11 ± 1.6e-2 | 0.16 ± 1.1e-2 | 1.6e-2 ± 1.4e-2 | 2.0e-3 ± 7.6e-4 | 4.9e-5 ± 8.4e-6 | **1.5e-4 ± 2.0e-5*** | 1.7e-5 ± 1.9e-6 | 1.3e-5 ± 6.8e-6 |
| Insulin degrading enzyme | IDE | 2.3e-2 ± 6.0e-4 | 2.3e-2 ± 9.0e-4 | 2.9e-2 ± 1.8e-3 | 2.5e-2 ± 2.9e-3 | 4.3e-3 ± 4.1e-4 | **9.4e-3 ± 9.2e-4*** | 4.5e-3 ± 8.8e-5 | 4.4e-3 ± 4.7e-4 |
| Membrane metalloprotease (neprilysin, NEP) | MME | 0.17 ± 1.8e-2 | 0.18 ± 1.1e-2 | 1.1e-2 ± 8.6e-3 | 1.0e-3 ± 4.3e-4 | 7.6e-3 ± 7.5e-3 | 1.5e-4 ± 4.2e-5 | 2.5e-4 ± 5.8e-5 | 8.6e-5 ± 1.9e-5 |
| ***Inflammatory cytokines and other growth factors*** |  |  |  |  |  |  |  |  |  |
| Tumor Necrosis Factor-alpha | TNF | 1.7e-4 ± 6.0e-5 | **6.1e-4 ± 1.0e-4*** | 2.2e-3 ± 7.9e-4 | 4.0e-3 ± 1.5e-3 | 2.8e-5 ± 1.1e-5 | **6.2e-4 ± 1.9e-4*** | 1.7e-5 ± 6.8e-6 | **6.0e-5 ± 9.2e-6*** |
| Interleukin-6 | IL6 | 3.0e-4 ± 2.1e-5 | **7.4e-3 ± 2.0e-3*** | 1.3e-3 ± 4.0e-4 | 3.4e-3 ± 1.3e-3 | 2.7e-5 ± 1.3e-5 | **7.9e-3 ± 1.9e-3*** | 2.1e-5 ± 7.4e-6 | **8.4e-4 ± 1.7e-4*** |
| Glycoprotein 130 | IL6ST | 6.5e-2 ± 6.2e-3 | **0.12 ± 6.2e-3*** | 0.12 ± 1.4e-2 | 0.14 ± 1.2e-2 | 1.3e-2 ± 3.0e-3 | **4.1e-2 ± 3.4e-3*** | 9.2e-3 ± 8.7e-4 | **1.6e-2 ± 2.5e-3*** |
| Leukemia inhibitory factor | LIF | 3.9e-4 ± 1.2e-4 | **9.1e-3 ± 3.0e-3*** | 1.7e-3 ± 2.0e-4 | **7.3e-2 ± 2.2e-2*** | 4.2e-5 ± 3.4e-6 | **1.9e-3 ± 6.7e-4*** | 2.5e-5 ± 1.0e-5 | **9.6e-5 ± 1.5e-5*** |
| Endothlin-1 | EDN1 | 1.6e-3 ± 3.5e-4 | 5.5e-3 ± 1.2e-3 | 8.5e-3 ± 4.8e-3 | 1.2e-2 ± 2.1e-3 | 5.0e-4 ± 1.4e-4 | **4.8e-3 ± 1.7e-3*** | 3.9e-4 ± 1.4e-4 | 5.1e-4 ± 7.4e-5 |
| Monocyte chemoattractant protein-1, MCP-1 | CCL2 | 1.6e-2 ± 8.0e-3 | **0.28 ± 6.3e-2*** | 0.25 ± 0.10 | **2.6 ± 0.73*** | 1.9e-3 ± 1.3e-3 | **2.8e-1 ± 7.9e-2*** | 1.8e-3 ± 1.0e-3 | **4.9e-2 ± 8.6e-3*** |
| Chemokine receptor 2 | CCR2 | 5.9e-4 ± 7.2e-5 | 4.6e-4 ± 2.8e-5 | 1.9e-3 ± 1.1e-3 | 8.3e-4 ± 2.0e-4 | 6.8e-5 ± 2.7e-5 | 3.0e-4 ± 8.0e-5 | 3.0e-5 ± 6.3e-6 | 3.6e-5 ± 1.6e-5 |
| Interleukin-1 beta | IL1B | 4.4e-4 ± 5.8e-5 | **9.2e-3 ± 4.8e-4*** | 3.5e-3 ± 1.7e-4 | 1.9e-3 ± 5.8e-4 | 5.9e-5 ± 4.8e-5 | **6.0e-4 ± 1.9e-4*** | 2.5e-5 ± 1.6e-5 | 1.2e-4 ± 2.7e-5 |
| Interleukin-10 | IL10 | 8.6e-5 ± 1.4e-5 | 7.8e-5 ± 1.4e-5 | 6.1e-4 ± 1.6e-4 | 2.6e-4 ± 4.7e-5 | 3.1e-5 ± 2.9e-6 | **1.3e-4 ± 3.8e-5*** | 1.8e-5 ± 4.3e-6 | 9.3e-6 ± 6.8e-6 |
| Interleukin-13 | IL13 | 1.4e-3 ± 1.9e-4 | 2.0e-3 ± 1.9e-4 | 3.2e-3 ± 6.9e-4 | 2.6e-3 ± 5.6e-4 | 3.8e-5 ± 1.3e-5 | **3.0e-4 ± 6.5e-5*** | 3.4e-5 ± 1.2e-5 | 5.3e-5 ± 1.4e-5 |
| Interferon-gamma | IFNG | 3.1e-4 ± 4.8e-5 | **1.3e-3 ± 2.4e-4*** | 4.5e-4 ± 5.6e-5 | **2.1e-3 ± 5.7e-4*** | 3.4e-5 ± 1.0e-5 | **7.4e-4 ± 1.1e-4*** | 1.3e-5 ± 3.2e-6 | **8.2e-5 ± 2.4e-5*** |
| Nerve growth factor receptor | NGFR | 7.1e-4 ± 7.0e-5 | 8.5e-4 ± 6.1e-5 | 2.3e-3 ± 7.3e-4 | 1.7e-3 ± 3.6e-4 | 5.0e-4 ± 6.2e-5 | **1.2e-3 ± 2.2e-4*** | 3.9e-4 ± 5.0e-5 | 4.3e-4 ± 4.5e-5 |
| Colony stimulating factor 3 | CSF3 | 1.2e-4 ± 1.6e-5 | **9.0e-4 ± 2.3e-4*** | 2.9e-4 ± 2.7e-5 | **8.0e-4 ± 1.2e-4*** | 9.0e-6 ± 1.98e-6 | **1.2e-3 ± 5.0e-4*** | 2.6e-6 ± 6.9e-7 | **1.3e-4 ± 3.8e-5*** |
| Hepatocyte growth factor | HGF | 4.6e-3 ± 9.1e-4 | 7.0e-3 ± 1.4e-3 | 1.3e-2 ± 4.0e-3 | 5.9e-3 ± 2.0e-3 | 4.2e-4 ± 1.5e-4 | 1.1e-3 ± 2.3e-4 | 1.6e-4 ± 4.7e-5 | 1.2e-4 ± 4.5e-5 |
| Epidermal growth factor | EGF | 7.3e-2 ± 1.1e-2 | 7.6e-2 ± 1.1e-2 | 0.13 ± 8.4e-2 | 9.0e-3 ± 3.1e-3 | 1.8e-3 ± 1.8e-4 | **6.7e-3 ± 9.9e-4*** | 5.2e-3 ± 9.8e-4 | 4.1e-3 ± 9.6e-4 |
| Platelet-Derived Growth Factor A | PDGFA | 3.3e-2 ± 2.9e-3 | 3.4e-2 ± 3.7e-3 | 8.6e-2 ± 2.2e-2 | 6.6e-2 ± 1.1e-2 | 2.1e-3 ± 2.3e-4 | **4.6e-3 ± 4.8e-4*** | 6.9e-4 ± 7.6e-5 | **9.6e-4 ± 4.2e-5*** |
| Platelet-Derived Growth Factor B | PDGFB | 4.0e-3 ± 4.3e-4 | **4.6e-2 ± 1.2e-2*** | 2.8e-2 ± 6.1e-3 | 6.6e-2 ± 1.8-e2 | 1.7e-3 ± 1.9e-4 | **4.0e-3 ± 3.9e-4*** | 1.4e-3 ± 5.6e-5 | 1.2e-3 ± 4.6e-4 |
| Platelet-Derived Growth Factor Receptor B | PDGFRB | 1.5e-3 ± 8.8e-5 | **2.4e-3 ± 2.2e-4*** | 7.2e-3 ± 1.4e-3 | 6.0e-3 ± 1.3e-3 | 9.3e-4 ± 4.6e-6 | **2.7e-3 ± 1.4e-4*** | 7.0e-4 ± 8.8e-5 | 9.4e-4 ± 2.0e-4 |
| Vascular endothelial growth factor A | VEGFA | 4.5e-2 ± 3.8e-3 | **7.9e-2 ± 1.2e-2*** | 8.0e-2 ± 1.9e-2 | 5.6e-2 ± 1.2e-2 | 2.0e-2 ± 1.9e-3 | 1.9e-2 ± 9.7e-4 | 1.9e-2 ± 1.8e-3 | 1.7e-2 ± 3.0e-3 |
| Vascular cell adhesion molecule-1 | VCAM1 | 3.4e-3 ±1.2e-3 | **0.12 ± 2.9e-2*** | 0.11 ± 4.0e-2 | **0.47 ± 0.12*** | 1.9e-3 ± 3.9e-4 | **6.3e-2 ± 1.7e-2*** | 1.1e-3 ± 2.6e-4 | **1.2e-2 ± 2.7e-3*** |
| Ras homolog gene family member A | RHOA | 0.15 ± 1.5e-2 | 0.17 ± 8.1e-3 | 0.40 ± 4.3e-2 | 0.50 ± 6.1e-2 | 2.7e-2 ± 2.2e-3 | **7.9e-2 ± 4.2e-3*** | 1.8e-2 ± 3.1e-3 | 2.3e-2 ± 3.1e-3 |
| Rho-associated coiled-coil containing protein-1 | ROCK1 | 1.9e-2 ± 6.0e-4 | 2.2e-2 ± 1.4e-3 | 4.0e-2 ± 3.4e-3 | 3.5e-2 ± 3.4e-3 | 3.1e-3 ± 1.4e-4 | **8.7e-3 ± 1.2e-3*** | 2.0e-3 ± 1.3e-4 | 2.6e-3 ± 3.3e-4 |
| Rho-associated coiled-coil containing protein-2 | ROCK2 | 2.7e-2 ± 3.3e-3 | 3.1e-2 ± 1.7e-3 | 0.10 ± 1.5e-2 | 0.12 ± 1.8e-2 | 8.9e-3 ± 6.9e-4 | **2.1e-2 ± 1.4e-3*** | 7.9e-3 ± 7.8e-5 | **1.2e-2 ± 1.1e-3*** |
| C-reactive protein | CRP | 8.7e-5 ± 0.2e-5 | **6.2e-4 ± 1.6e-4*** | 3.7e-5 ± 6.5e-6 | 4.8e-5 ± 1.0e-5 | 1.9e-4 ± 1.8e-4 | 3.3e-6 ± 2.5e-7 | 4.1e-7 ± 1.4e-7 | 1.3e-6 ± 6.6e-7 |
| Insulin | INS | 8.0e-4 ± 6.3e-5 | 1.0e-3 ± 6.0e-5 | 1.4e-3 ± 2.8e-4 | 1.5e-3 ± 4.1e-4 | 2.6e-5 ± 7.5e-6 | **1.8e-4 ± 3.0e-5*** | 1.2e-5 ± 3.1e-6 | 3.3e-5 ± 9.9e-6 |
| Von Willebrand factor | VWF | 1.4e-3 ± 1.4e-4 | 1.4e-3 ± 1.0e-4 | 6.0e-3 ± 2.0e-3 | 3.7e-3 ± 1.7e-3 | 9.2e-3 ± 1.1e-3 | **4.0e-2 ± 5.1e-3*** | 1.1e-2 ± 5.9e-4 | 1.5e-2 ± 1.8e-3 |
| Tissue plasminogen activator | PLAT | 1.9e-2 ± 1.5e-3 | **7.6e-2 ± 5.5e-3*** | 4.4e-2 ± 2.4e-3 | **7.9e-2 ± 1.3e-2*** | 9.2e-4 ± 1.7e-4 | **1.3e-2 ± 1.2e-3*** | 2.4e-4 ± 2.5e-5 | **7.4e-4 ± 1.2e-4*** |
| Plasminogen | PLG | 1.3e-5 ± 1.9e-6 | 1.5e-5 ± 4.7e-6 | 5.3e-5 ± 2.4e-5 | 6.4e-5 ± 2.7e-5 | 1.0e-4 ± 1.2e-5 | 7.0e-5 ± 2.1e-5 | 3.7e-5 ± 6.0e-6 | 4.9e-5 ± 1.2e-5 |
| Plasminogen activator inhibitor-1 | SERPINE1 | 3.0e-3 ± 1.5e-3 | **7.9e-2 ± 1.6e-2*** | 1.1e-2 ± 3.1e-3 | **1.4e-1 ± 2.9e-2*** | 8.4e-4 ± 3.1e-4 | **7.3e-2 ± 1.2e-2*** | 6.5e-4 ± 2.4e-4 | **1.9e-2 ± 2.5e-3*** |
| Adiponectin | ADIPOQ | 6.8e-5 ± 2.4e-5 | 5.3e-5 ± 1.2e-5 | 9.1e-4 ± 4.0e-4 | 1.6e-2 ± 1.5-e2 | 1.3e-2 ± 5.1e-3 | 4.7e-3 ± 2.3e-3 | 7.3e-4 ± 6.8e-4 | 5.3e-5 ± 1.9e-5 |
| Mitogen-activated protein kinase 1 | MAPK1 | 2.9e-2 ± 2.3e-3 | 3.2e-2 ± 1.0e-3 | 7.2e-2 ± 5.6e-3 | 6.6e-2 ± 7.7e-3 | 6.0e-3 ± 6.2e-4 | **1.3e-2 ± 1.2e-3*** | 3.6e-3 ± 2.2e-4 | 4.0e-3 ± 5.2e-4 |
| RAC-alpha serine/threonine-protein kinase 1 | AKT1 | 2.3e-2 ± 8.6e-4 | 2.4e-2 ± 1.6e-3 | 6.1e-2 ± 7.1e-3 | 6.0e-2 ± 1.0e-2 | 5.5e-3 ± 3.5e-4 | **1.4e-2 ± 7.0e-4*** | 4.9e-3 ± 2.1e-4 | 5.9e-3 ± 4.4e-4 |
| Signal transducer and activator of transcription 1 | STAT1 | 2.5e-2 ± 5.1e-3 | 3.7e-2 ± 2.4e-3 | 5.0e-2 ± 5.7e-3 | 4.5e-2 ± 1.1e-2 | 4.5e-3 ± 1.7e-3 | 1.0e-2 ± 1.3e-3 | 2.1e-3 ± 5.6e-4 | 2.4e-3 ± 6.9e-4 |
| Signal transducer and activator of transcription 3 | STAT3 | 3.7e-2 ± 1.0e-2 | **7.4e-2 ± 4.1e-3*** | 6.4e-2 ± 1.1e-2 | 9.3e-2 ± 1.9e-2 | 9.7e-3 ± 2.9e-3 | **2.6e-2 ± 1.7e-3*** | 4.4e-3 ± 8.1e-4 | 6.6e-3 ± 1.4e-3 |
| Nuclear factor kappa-B | NFKB1 | 7.0e-3 ± 4.4e-4 | **3.8e-2 ± 6.7e-3*** | 2.6e-2 ± 1.6e-3 | **6.5e-2 ± 7.2e-3*** | 2.2e-3 ± 2.4e-4 | **1.6e-2 ± 2.3e-3*** | 1.5e-3 ± 1.1e-4 | **5.8e-3 ± 7.4e-4*** |
| Transcription factor Sp1 | SP1 | 1.1e-2 ± 1.2e-3 | 1.1e-2 ± 5.6e-4 | 3.5e-2 ± 7.9e-3 | 2.5e-2 ± 4.7e-3 | 1.9e-3 ± 2.5e-4 | **3.0e-3 ± 1.9e-4*** | 1.3e-3 ± 1.4e-4 | 1.2e-3 ± 2.2e-4 |
| ***Renal inflammation and injury*** |  |  |  |  |  |  |  |  |  |
| Interleukin-1 alpha | IL1A | 4.8e-4 ± 1.3e-4 | **3.5e-3 ± 8.6e-4*** | 2.3e-3 ± 4.6e-4 | **7.9e-3 ± 1.5-e3*** | - | - | - | - |
| Interleukin-7 | IL7 | 3.7e-3 ± 3.2e-4 | 3.7e-3 ± 3.7e-4 | 3.3e-3 ± 4.9e-4 | 3.6e-3 ± 7.0e-4 | - | - | - | - |
| Interleukin-8 | IL8 | 2.6e-4 ± 6.9e-5 | **9.8e-3 ± 3.2e-3*** | 1.3e-3 ± 4.6e-4 | **2.1e-2 ± 7.9-e3*** | - | - | - | - |
| Interleukin-18 | IL18 | 1.6e-3 ± 1.5e-4 | **6.1e-3 ± 1.0e-3*** | 4.7e-3 ± 1.2e-3 | 7.1e-3 ± 2.7e-3 | - | - | - | - |
| Granulocyte Macrophage colony-stimulating Factor | CSF2 | 3.0e-4 ± 3.9e-5 | 4.2e-4 ± 1.4e-4 | 9.1e-5 ± 1.8e-5 | 2.3e-4 ± 6.7e-5 | - | - | - | - |
| granulocyte-colony stimulating factor | CSF3 | 1.2e-4 ± 2.4e-5 | **9.7e-4 ± 2.8e-4*** | 3.0e-4 ± 7.1e-5 | 9.1e-4 ± 1.6e-4 | - | - | - | - |
| Insulin-like growth factor 1 | IGF1 | 2.8e-3 ± 4.9e-4 | 2.1e-3 ± 1.4e-4 | 3.4e-2 ± 1.0e-2 | 1.6e-2 ± 3.2e-3 | - | - | - | - |
| Neural cell adhesion molecule 1 | NCAM1 | 3.6e-4 ± 4.8e-5 | 4.7e-4 ± 6.2e-5 | 3.9e-3 ± 1.3e-3 | 2.6e-3 ± 1.7e-4 | - | - | - | - |
| Intercellular Adhesion Molecule 1 | ICAM1 | 1.9e-3 ± 5.4e-4 | **0.11 ± 2.2e-2*** | 2.6e-2 ± 9.4e-3 | **0.39 ± 6.8e-2*** | - | - | - | - |
| P-selectin | SELP | 8.0e-4 ± 7.7e-5 | **2.3e-3 ± 3.2e-4*** | 1.2e-3 ± 7.5e-4 | 9.1e-4 ± 4.2e-4 | - | - | - | - |
| E-selectin | SELE | 7.8e-4 ± 4.3e-4 | **0.11 ± 3.3e-2*** | 1.6e-3 ± 6.5e-4 | **8.4e-2 ± 3.1-e2*** | - | - | - | - |
| Chemokine (C-C motif) ligand 5, RANTES | CCL5 | 9.2e-5 ± 1.0e-5 | 2.8e-4 ± 9.0e-5 | 1.4e-3 ± 1.0e-3 | 4.7e-4 ± 3.2e-4 | - | - | - | - |
| C-C chemokine receptor type 1 | CCR1 | 1.1e-4 ± 2.6e-5 | 2.1e-4 ± 4.6e-5 | 3.3e-4 ± 7.7e-5 | 3.6e-4 ± 1.3e-4 | - | - | - | - |
| C-C chemokine receptor type 3 | CCR3 | 1.4e-5 ± 4.0e-6 | 8.9e-6 ± 2.9e-6 | 3.5e-5 ± 2.5e-5 | 7.5e-6 ± 5.4e-6 | - | - | - | - |
| Serum amyloid A1 | SAA1 | 1.2e-4 ± 2.4e-5 | **1.7e-3 ± 6.9e-4*** | 4.9e-3 ± 2.1e-3 | 7.1e-3 ± 2.0e-3 | - | - | - | - |
| N-acetyl-beta-(D)-glucosaminidase, NAG | MGEA5 | 3.2e-2 ± 3.2e-3 | 3.5e-2 ± 1.2e-3 | 5.2e-2 ± 8.0e-3 | **0.12 ± 1.8e-2*** | - | - | - | - |
| kidney injury molecule-1, KIM-1 hepatitis A virus cellular receptor1 | HAVCR1 | 1.2e-3 ± 1.6e-4 | 2.3e-3 ± 4.2e-4 | 3.6e-3 ± 8.6e-4 | 3.3e-3 ± 1.1e-3 | - | - | - | - |
| Annexin A5 | ANXA5 | 6.6e-1 ± 7.1e-3 | 7.3e-2 ± 3.8e-3 | 0.40 ± 5.6e-2 | 0.23 ± 5.1e-2 | - | - | - | - |
| Thrombospondin 1, TSP-1 | THBS1 | 7.0e-2 ± 4.0e-3 | 7.6e-2 ± 1.3e-2 | 0.13 ± 1.7e-2 | 0.15 ± 4.1e-2 | - | - | - | - |
| Calponin 1 | CNN1 | 2.1e-3 ± 3.0e-4 | **5.1e-3 ± 2.4e-4*** | 1.2e-2 ± 9.7e-3 | 1.3e-2 ± 1.1e-2 | - | - | - | - |
| Fibulin-1 | FBLN1 | 6.4e-4 ± 1.1e-4 | 8.8e-4 ± 7.7e-5 | 2.7e-2 ± 7.7e-3 | 2.0e-2 ± 5.2e-3 | - | - | - | - |
| Ankyrin1 | ANK1 | 3.2e-4 ± 4.4e-5 | 3.0e-4 ± 4.9e-5 | 1.5e-3 ± 3.0e-4 | 6.7e-4 ± 1.8e-4 | - | - | - | - |
| Spectrin1 | SPTA1 | 4.0e-6 ± 1.1e-6 | 4.5e-6 ± 1.2e-6 | 3.3e-5 ± 1.0e-5 | 2.9e-5 ± 1.3e-5 | - | - | - | - |
| Heat shock protein 27 | HSPB1 | 0.11 ± 1.9e-2 | 0.17 ± 2.6e-2 | 0.24 ± 1.8e-2 | 0.32 ± 3.3e-2 | - | - | - | - |
| Toll-like receptor 2 | TLR2 | 2.2e-4 ± 2.4e-5 | 2.6e-4 ± 3.8e-5 | 1.1e-3 ± 2.3e-4 | 7.4e-4 ± 9.4e-5 | - | - | - | - |
| S100 calcium-binding protein A4 | S100A4 | 1.1e-3 ± 1.0e-4 | 1.6e-3 ± 2.4e-4 | 1.9e-2 ± 1.0e-2 | 5.9e-3 ± 1.6e-3 | - | - | - | - |
| S100 calcium-binding protein A6 | S100A6 | 2.2e-2 ± 4.6e-3 | 2.4e-2 ± 3.6e-3 | 0.38 ± 2.5e-2 | 0.49 ± 9.5e-2 | - | - | - | - |
| Clusterin, apolipopritein J | CLU | 2.0e-2 ± 6.4e-3 | 4.4e-2 ± 9.3e-3 | 0.14 ± 3.0e-2 | 0.25 ± 4.8e-2 | - | - | - | - |
| Glutathione S-transferase alpha3 | GSTA3 | 8.2e-3 ± 5.6e-3 | 3.1e-3 ± 9.4e-4 | 4.3e-3 ± 1.3e-3 | 1.5e-2 ± 1.4e-2 | - | - | - | - |
| neutrophil gelatinase-associated lipoprotein  (NGAL), Lipocalin 2 (LCN2) | LCN2 | 1.6e-4 ± 1.6e-5 | 1.5e-4 ± 1.0e-5 | 4.4e-4 ± 5.1e-5 | 4.1e-4 ± 1.0e-4 | - | - | - | - |
| Trefoil factor 3 | TFF3 | 9.3e-5 ± 2.0e-5 | 1.4e-4 ± 3.6e-5 | 1.9e-4 ± 2.5e-5 | 4.0e-4 ± 1.3e-4 | - | - | - | - |
| FoxP3, forkhead box P3 | FOXP3 | 1.7e-3 ± 4.9e-4 | 1.8e-3 ± 1.1e-3 | 1.6e-3 ± 2.4e-4 | 4.5e-3 ± 2.3e-3 | - | - | - | - |
| Immediate early response 3 | IER3 | 6.8e-3 ± 8.6e-4 | **5.8e-2 ± 1.6e-2*** | 2.7e-2 ± 3.3e-3 | **7.9e-2 ± 1.3-e2*** | - | - | - | - |
| Signal transducer and activator of transcription 2 | STAT2 | 1.2e-2 ± 1.4e-3 | **1.8e-2 ± 9.0e-4*** | 3.1e-2 ± 2.9e-3 | 2.3e-2 ± 1.8e-3 | - | - | - | - |
| Matrix Gla protein | MGP | 6.9e-5 ± 1.4e-5 | 1.3e-4 ± 2.2e-5 | 2.0e-4 ± 3.0e-5 | 2.1e-4 ± 6.8e-5 | - | - | - | - |
| Superoxide dismutase 1 | SOD1 | 0.19 ± 1.4e-2 | 0.17 ± 7.1e-3 | 0.13 ± 1.4e-2 | 0.12 ± 1.7e-2 | - | - | - | - |
| Catalase | CAT | 0.50 ± 3.2e-2 | 0.44 ± 2.2e-2 | 0.22 ± 7.5e-2 | 8.9e-2 ± 1.4e-2 | - | - | - | - |
| Na, K-ATPase1 | ATP1A1 | 0.41 ± 3.3e-2 | 0.53 ± 3.8e-2 | 0.84 ± 0.40 | 0.30 ± 2.5e-2 | - | - | - | - |
| Na, K-ATPase2 | ATP1A2 | 1.3e-3 ± 1.4e-4 | 1.7e-3 ± 2.8e-4 | 1.4e-2 ± 3.9e-3 | 2.0e-2 ± 3.7e-3 | - | - | - | - |
| Lysyl oxidase | LOX | 7.1e-4 ± 1.8e-4 | 7.5e-4 ± 6.1e-5 | 1.6e-2 ± 3.8e-3 | 7.0e-3 ± 1.3e-3 | - | - | - | - |
| Kruppel-like transcription factor, Zf9 | KLF6 | 6.2e-3 ± 8.8e-4 | **2.2e-2 ± 3.8e-3*** | 1.8e-2 ± 3.9e-3 | **4.3e-2 ± 3.9e-3*** | - | - | - | - |
| C4 component | C4A | 2.6e-3 ± 1.4e-3 | 3.6e-3 ± 1.1e-3 | 7.2e-4 ± 1.1e-4 | 1.9e-3 ± 9.1e-4 | - | - | - | - |
| ***Apoptosis*** |  |  |  |  |  |  |  |  |  |
| Cytochrome c-1 | CYC1 | 7.5e-2 ± 6.0e-3 | 7.7e-2 ± 3.4e-3 | 7.8e-2 ± 2.4e-2 | 3.8e-2 ± 4.7e-3 | 4.9e-2 ± 3.9e-3 | 5.1e-2 ± 3.0e-3 | 5.0e-2 ± 2.1e-3 | 4.1e-2 ± 5.0e-3 |
| Apoptotic protease activating factor 1 | APAF1 | 3.6e-3 ± 3.7e-4 | 2.9e-3 ± 1.8e-4 | 1.2e-2 ± 6.1e-4 | **9.5e-3 ± 1.1e-3*** | 7.9e-4 ± 1.2e-4 | **1.6e-3 ± 1.7e-4*** | 4.0e-4 ± 2.7e-5 | 4.3e-4 ± 8.4e-5 |
| B-cell lymphoma 2 associated X protein | BAX | 7.9e-3 ± 5.2e-4 | 8.3e-3 ± 7.6e-4 | 1.5e-2 ± 2.3e-3 | 1.0e-2 ± 1.7e-3 | 9.1e-4 ± 3.4e-4 | 1.8e-3 ± 2.9e-4 | 3.9e-4 ± 3.5e-5 | 3.9e-4 ± 6.8e-5 |
| B-cell lymphoma 2 | BCL2 | 1.1e-2 ± 1.1e-3 | 1.3e-2 ± 8.4e-4 | 2.8e-2 ± 6.8e-3 | 3.2e-2 ± 5.4e-3 | 2.1e-3 ± 6.2e-4 | 3.9e-3 ± 4.2e-4 | 1.1e-3 ± 1.1e-4 | 1.5e-3 ± 2.0e-4 |
| B-cell lymphoma 2 associated X protein | BAX | 7.9e-3 ± 5.2e-4 | 8.3e-3 ± 7.6e-4 | 1.5e-2 ± 2.3e-3 | 1.0e-2 ± 1.7e-3 | 9.1e-4 ± 3.4e-4 | 1.8e-3 ± 2.9e-4 | 3.9e-4 ± 3.5e-5 | 3.9e-4 ± 6.8e-5 |
| Bcl-2 associated death promoter | BAD | 2.8e-3 ± 2.3e-4 | 2.7e-3 ± 2.6e-4 | 5.1e-3 ± 5.8e-4 | 3.2e-3 ± 5.4e-4 | 2.0e-3 ± 3.8e-4 | 1.3e-3 ± 1.6e-4 | 4.6e-4 ± 9.0e-5 | 4.1e-4 ± 5.8e-5 |
| BCL2-antagonist/killer 1 | BAK1 | 4.8e-3 ± 1.1e-3 | 4.9e-3 ± 5.3e-4 | 1.2e-2 ± 1.4e-3 | 1.4e-2 ± 3.5e-3 | 7.9e-4 ± 2.3e-4 | 1.9e-3 ± 2.6e-4 | 5.1e-4 ± 5.6e-5 | 5.2e-4 ± 1.9e-4 |
| BH3 interacting-domain death agonist | BID | 3.1e-2 ± 3.0e-3 | 3.2e-2 ± 1.5e-3 | 4.2e-2 ± 6.3e-3 | 2.8e-2 ± 4.2e-3 | 5.4e-3 ± 1.2e-3 | 7.9e-3 ± 5.3e-4 | 3.0e-3 ± 2.2e-4 | 3.4e-3 ± 3.8e-4 |
| Baculoviral IAP repeat containing 2 | BIRC2 | 1.3e-2 ± 6.3e-4 | **5.4e-2 ± 1.2e-2*** | 4.2e-2 ± 5.7e-3 | **9.3e-2 ± 1.5e-2*** | 3.0e-3 ± 4.5e-4 | **9.3e-3 ± 1.1e-3*** | 2.0e-3 ± 7.0e-5 | **3.6e-3 ± 3.8e-4*** |
| Baculoviral IAP repeat containing 3 | BIRC3 | 5.4e-3 ± 2.3e-3 | **0.14 ± 2.3e-2*** | 2.5e-2 ± 6.4e-3 | **0.37 ± 7.2e-2*** | 1.9e-3 ± 8.8e-4 | **4.8e-2 ± 6.5e-3*** | 1.5e-3 ± 6.1e-4 | **2.3e-2 ± 2.2e-3*** |
| Caspase 2 | CASP2 | 2.3e-3 ± 1.6e-4 | 3.1e-3 ± 2.0e-4 | 6.8e-3 ± 6.2e-4 | **1.0e-2 ± 1.2e-3*** | 5.3e-4 ± 1.1e-4 | **1.2e-3 ± 7.0e-5*** | 2.8e-4 ± 1.1e-5 | 4.0e-4 ± 7.8e-5 |
| Caspase 3 | CASP3 | 8.3e-3 ± 1.2e-3 | 9.4e-3 ± 3.2e-4 | 1.4e-2 ± 3.5e-3 | 1.0e-2 ± 1.7-e3 | 1.9e-3 ± 3.2e-4 | **4.8e-3 ± 1.7e-4*** | 2.0e-3 ± 3.3e-4 | 3.2e-3 ± 3.6e-4 |
| Caspase 7 | CASP7 | 3.8e-3 ± 7.2e-4 | 4.2e-3 ± 5.0e-4 | 6.5e-3 ± 8.9e-4 | 9.2e-3 ± 1.9e-3 | 8.4e-4 ± 3.0e-4 | **2.0e-3 ± 1.7e-4*** | 4.5e-4 ± 5.5e-5 | 7.5e-4 ± 3.2e-4 |
| Caspase 8 | CASP8 | 6.3e-3 ± 6.3e-4 | 5.4e-3 ± 7.0e-4 | 3.1e-3 ± 9.7e-4 | 3.0e-3 ± 1.0e-3 | 6.6e-4 ± 3.2e-4 | 1.4e-3 ± 1.6e-4 | 3.5e-4 ± 8.4e-5 | 9.1e-4 ± 2.4e-4 |
| Caspase 9 | CASP9 | 7.8e-3 ± 9.6e-4 | 7.7e-3 ± 6.9e-4 | 1.5e-2 ± 1.1e-3 | 1.3e-2 ± 1.2e-3 | 1.5e-3 ± 2.0e-4 | **3.3e-3 ± 2.8e-4*** | 1.1e-3 ± 3.6e-5 | 1.1e-3 ± 1.4e-4 |
| CASP and FADD like apoptosis regulator | CFLAR | 3.4e-2 ± 2.3e-3 | **6.6e-2 ± 1.0e-2*** | 9.4e-2 ± 1.2e-2 | 0.17 ± 3.1e-2 | 6.7e-3 ± 1.7e-3 | **1.8e-2 ± 2.9e-3*** | 3.6e-3 ± 3.3e-4 | 6.6e-3 ± 1.2e-3 |
| Conserved helix-loop-helix ubiquitous kinase | CHUK | 1.1e-2 ± 9.2e-4 | 1.2e-2 ± 9.2e-4 | 1.3e-2 ± 9.2e-4 | 1.4e-2 ± 1.9e-3 | 1.2e-3 ± 3.9e-4 | 2.0e-3 ± 1.6e-4 | 5.3e-4 ± 2.8e-5 | 8.2e-4 ± 1.0e-4 |
| Death-associated protein 6 | DAXX | 2.4e-3 ± 1.6e-4 | 2.0e-3 ± 1.7e-4 | 7.3e-3 ± 6.2e-4 | 6.5e-3 ± 1.3e-3 | 6.1e-4 ± 7.0e-5 | 8.4e-4 ± 7.6e-5 | 2.8e-4 ± 1.8e-5 | 3.0e-4 ± 4.1e-5 |
| Diablo homolog, mitochondrial | DIABLO | 1.1e-2 ± 1.8e-3 | 1.2e-2 ± 1.3e-3 | 1.3e-2 ± 2.0e-3 | 1.6e-2 ± 2.1e-3 | 2.5e-3 ± 7.5e-4 | 4.7e-3 ± 6.0e-4 | 1.3e-3 ± 8.7e-5 | 1.6e-3 ± 2.2e-4 |
| Calcineruin A alpha | PPP3CA | 5.7e-3 ± 6.4e-4 | 6.6e-3 ± 5.1e-4 | 1.7e-2 ± 1.8e-3 | 1.1e-2 ± 1.7e-3 | 2.0e-3 ± 2.5e-4 | **5.6e-3 ± 6.7e-4*** | 1.2e-3 ± 6.4e-5 | 1.3e-3 ± 1.8e-4 |
| Fas (TNF superfamily member 6) | FAS | 2.4e-3 ± 4.6e-4 | **1.0e-2 ± 6.5e-4*** | 1.6e-2 ± 3.1e-3 | 2.7e-2 ± 4.4-e3 | 4.1e-4 ± 9.0e-5 | **3.3e-3 ± 3.6e-4*** | 1.9e-4 ± 4.6e-5 | **5.2e-4 ± 1.5e-4*** |
| Fas Ligand | FASLG | 1.8e-4 ± 3.9e-5 | 2.9e-4 ± 7.8e-5 | 4.8e-4 ± 1.1e-4 | 5.4e-4 ± 2.6e-4 | 1.2e-5 ± 4.4e-6 | **5.2e-5 ± 1.3e-5*** | 7.4e-6 ± 1.5e-6 | 1.5e-5 ± 5.2e-6 |
| TNF receptor superfamily member 12A | TNFRSF12A | 1.7e-3 ± 3.2e-4 | **7.6e-3 ± 1.9e-3*** | 1.0e-2 ± 3.7e-3 | **7.3e-2 ± 1.6e-2*** | 9.5e-4 ± 9.0e-5 | **1.0e-2 ± 1.6e-3*** | 1.4e-3 ± 2.9e-4 | 2.0e-3 ± 1.3e-4 |
| TNF receptor superfamily member 1A | TNFRSF1A | 5.2e-3 ± 1.6e-3 | **1.2e-2 ± 3.8e-4*** | 1.2e-2 ± 2.0e-3 | 1.7e-2 ± 3.9e-3 | 1.5e-3 ± 5.1e-4 | **4.7e-3 ± 4.4e-4*** | 5.4e-4 ± 1.2e-4 | 8.6e-4 ± 1.8e-4 |
| TNF receptor superfamily member 21 | TNFRSF21 | 2.3e-2 ± 5.2e-3 | 3.1e-2 ± 3.3e-3 | 0.15 ± 2.7e-2 | 0.16 ± 1.8e-2 | 3.2e-3 ± 1.1e-3 | 6.4e-3 ± 3.9e-4 | 1.3e-3 ± 1.0e-4 | 1.5e-3 ± 3.1e-4 |
| TNF receptor superfamily member 25 | TNFRSF25 | 7.1e-4 ± 2.5e-4 | 4.3e-4 ± 4.7e-5 | 1.8e-3 ± 7.1e-4 | 1.1e-3 ± 4.4e-4 | 2.6e-4 ± 5.3e-5 | 5.7e-4 ± 7.5e-5 | 1.8e-4 ± 1.5e-5 | 1.3e-4 ± 2.5e-5 |
| TNF superfamily member 10 | TNFSF10 | 5.3e-2 ± 9.3e-3 | **0.15 ± 2.1e-2*** | 0.21 ± 5.1e-2 | 0.28 ± 5.6e-3 | 1.7e-2 ± 6.0e-3 | 2.7e-2 ± 3.3e-3 | 9.9e-3 ± 2.6e-3 | 9.4e-3 ± 2.5e-3 |
| lymphotoxin alpha (TNF superfamily member1) | LTA | 9.7e-6 ± 1.0e-6 | **4.2e-5 ± 1.1e-5*** | 1.6e-4 ± 5.4e-5 | 2.3e-4 ± 8.4e-5 | 1.7e-6 ± 7.0e-7 | 1.5e-5 ± 5.5e-6 | 8.9e-7 ± 3.8e-7 | 3.3e-6 ± 9.8e-7 |
| Inhibitor of kappa light polypeptide B gene enhancer in B-cells, kinase beta | IKBKB | 1.0e-2 ± 1.0e-3 | **3.8e-2 ± 3.9e-3*** | 2.1e-2 ± 1.6e-3 | 4.2e-2 ± 6.2e-3 | 1.8e-3 ± 4.9e-4 | **5.3e-3 ± 5.2e-4*** | 6.1e-4 ± 4.1e-5 | **1.5e-3 ± 1.5e-4*** |
| Inhibitor of kappa light polypeptide G gene enhancer in B-cells,kinase gamma | IKBKG | 3.3e-3 ± 4.6e-4 | 3.1e-3 ± 2.7e-4 | 9.8e-3 ± 1.1e-3 | 8.2e-3 ± 1.0e-3 | 1.0e-3 ± 1.4e-4 | 1.6e-3 ± 1.4e-4 | 6.4e-4 ± 7.8e-5 | 6.5e-4 ± 1.0e-4 |
| Apoptosis signal-regulating kinase-1  (ASK1=MAP3K5) | MAP3K5 | 9.3e-3 ± 1.5e-3 | 1.5e-2 ± 2.4e-3 | 2.3e-2 ± 1.8e-3 | **3.4e-2 ± 1.5e-3*** | 1.4e-3 ± 3.2e-4 | **3.3e-3 ± 3.2e-4*** | 3.1e-3 ± 3.1e-4 | 3.7e-3 ± 5.5e-4 |
| Mitogen-activated protein kinase 8 | MAPK8 | 2.3e-2 ± 2.2e-3 | 2.7e-2 ± 2.1e-3 | 6.4e-2 ± 4.1e-3 | 8.0e-2 ± 1.2e-2 | 5.8e-3 ± 7.5e-4 | 9.9e-3 ± 1.3e-3 | 2.8e-3 ± 1.3e-4 | 3.6e-3 ± 3.3e-4 |
| Mitogen-activated protein kinase 9 | MAPK9 | 8.5e-3 ± 6.5e-4 | 8.4e-3 ± 4.7e-4 | 2.7e-2 ± 3.0e-3 | 2.0e-2 ± 3.4e-3 | 3.2e-3 ± 2.4e-4 | **5.1e-3 ± 3.1e-4*** | 2.7e-3 ± 9.3e-5 | 2.8e-3 ± 3.7e-4 |
| Nucleotide-binding oligomerization domain  containing protein 1 | NOD1 | 2.4e-3 ± 2.0e-4 | 2.3e-3 ± 2.8e-4 | 9.8e-3 ± 1.5e-3 | 6.3e-3 ± 1.1e-3 | 5.8e-4 ± 2.6e-5 | **2.3e-3 ± 3.6e-4*** | 2.8e-4 ± 6.3e-6 | 3.6e-4 ± 6.5e-5 |
| Myc | MYC | 9.3e-3 ± 3.8e-3 | **7.2e-2 ± 1.7e-2*** | 1.3e-2 ± 2.2e-3 | **4.5e-2 ± 7.8-e3*** | 3.4e-3 ± 1.1e-3 | **1.6e-2 ± 1.3e-3*** | 1.3e-3 ± 2.8e-4 | **4.3e-3 ± 9.2e-4*** |
| Nerve growth factor | NGF | 2.1e-4 ± 3.3e-5 | 3.3e-4 ± 5.0e-5 | 1.6e-3 ± 3.8e-4 | 9.8e-4 ± 4.4e-4 | 1.2e-4 ± 1.4e-5 | 1.3e-4 ± 2.5e-5 | 9.8e-5 ± 1.0e-5 | 6.7e-5 ± 1.7e-5 |
| Nerve growth factor receptor | NGFR | 8.8e-4 ± 1.5e-4 | 1.2e-3 ± 2.4e-4 | 2.7e-3 ± 8.2e-4 | 2.5e-3 ± 7.7e-4 | 4.5e-4 ± 7.0e-5 | **1.2e-3 ± 2.1e-4*** | 3.4e-4 ± 5.4e-5 | 3.5e-4 ± 3.8e-5 |
| Poly (ADF-ribose) polumerase 1 | PARP1 | 7.93e-3 ± 8.5e-4 | 6.2e-3 ± 4.3e-4 | 2.3e-2 ± 1.7e-3 | **1.3e-2 ± 1.7e-3*** | 3.1e-3 ± 3.9e-4 | 4.5e-3 ± 2.6e-4 | 2.4e-3 ± 1.1e-4 | 2.4e-3 ± 3.4e-4 |
| Transcription factor p65 | RELA | 1.1e-2 ± 8.4e-4 | **2.5e-2 ± 2.0e-3*** | 3.6e-2 ± 3.2e-3 | **7.1e-2 ± 8.9e-3** | 3.2e-3 ± 4.6e-4 | **9.9e-3 ± 1.1e-3*** | 1.9e-3 ± 2.3e-5 | **4.2e-3 ± 5.8e-4*** |
| Calpain-2 | CAPN2 | 2.1e-2 ± 2.4e-3 | 2.3e-2 ± 2.2e-3 | 0.15 ± 4.5e-3 | 0.14 ± 2.2e-2 | 5.9e-3 ± 7.6e-4 | **1.7e-2 ± 1.6e-3*** | 3.7e-3 ± 4.7e-4 | 5.2e-3 ± 6.7e-4 |
| X-linked inhibitor of apoptosis | XIAP | 1.6e-2 ± 1.5e-3 | 1.7e-2 ± 7.0e-4 | 4.2e-2 ± 4.9e-3 | 3.2e-2 ± 3.5e-3 | 4.9e-3 ± 7.4e-4 | 7.6e-3 ± 7.2e-4 | 2.7e-3 ± 2.2e-4 | 2.9e-3 ± 3.6e-4 |
| Tumor protein p53 | TP53 | 4.9e-3 ± 5.3e-4 | **1.1e-2 ± 3.4e-4*** | 1.7e-2 ± 2.8e-3 | 2.6e-2 ± 3.6-e3 | 1.1e-3 ± 2.9e-4 | **4.0e-3 ± 4.1e-4*** | 5.9e-4 ± 5.0e-5 | **1.3e-3 ± 2.7e-4*** |
| Mitochondrial carnitine/acylcarnitine  carrier protein | SLC25A29 | 2.2e-4 ± 3.0e-5 | **4.4e-4 ± 4.6e-5*** | 1.8e-3 ± 3.2e-4 | 2.2e-3 ± 2.9e-4 | 6.1e-5 ± 2.0e-5 | **1.7e-4 ± 5.7e-6*** | 4.2e-5 ± 8.1e-6 | 8.5e-5 ± 2.8e-5 |
| ***Fibrosis*** |  |  |  |  |  |  |  |  |  |
| Collagen type I | COL1A1 | 2.7e-3 ± 5.5e-4 | **5.0e-3 ± 2.8e-4*** | 7.1e-2 ± 6.1e-2 | 2.7e-2 ± 1.7e-2 | 1.5e-2 ± 1.6e-3 | **9.3e-1 ± 1.6e-1*** | 3.4e-3 ± 7.2e-4 | 7.5e-3 ± 1.9e-3 |
| Collagen type III | COL3A1 | 3.4e-3 ± 9.1e-4 | 3.5e-3 ± 5.1e-4 | 0.11 ± 2.9e-2 | 5.7e-2 ± 7.4e-3 | 4.4e-2 ± 5.0e-4 | **7.1e-1 ± 1.2e-1*** | 1.3e-2 ± 3.0e-3 | 2.0e-2 ± 4.4e-3 |
| Collagen type IV | COL4A1 | 2.9e-2 ± 3.0e-3 | **4.9e-2 ± 3.5e-3*** | 8.5e-2 ± 2.0e-2 | 9.1e-2 ± 6.6e-3 | 1.2e-2 ± 1.1e-3 | **1.1e-1 ± 9.2e-3*** | 9.3e-4 ± 7.2e-4 | 2.0e-2 ± 7.0e-3 |
| Collagen type IV | COL4A2 | 3.3e-2 ± 3.4e-3 | **4.9e-2 ± 3.3e-3*** | 0.11 ± 2.2e-2 | 9.9e-2 ± 1.1e-2 | 1.5e-2 ± 1.6e-3 | **0.13 ± 1.2e-2*** | 1.2e-2 ± 1.2e-3 | 2.1e-2 ± 6.9e-3 |
| Collagen type XV | COL15A1 | 5.4e-3 ± 1.6e-3 | 7.2e-3 ± 6.9e-4 | 2.7e-2 ± 2.1e-2 | 6.9e-3 ± 3.7e-3 | 1.4e-2 ± 1.4e-3 | **4.8e-2 ± 6.6e-3*** | 7.7e-3 ± 1.3e-3 | 1.1e-2 ± 3.0e-3 |
| Contactin-1 | CNTN1 | 3.7e-5 ± 4.5e-6 | 5.7e-5 ± 1.3e-5 | 2.2e-3 ± 1.3e-3 | 6.0e-4 ± 3.0e-4 | 1.7e-4 ± 2.4e-5 | 1.2e-4 ± 5.1e-6 | 2.8e-5 ± 8.6e-6 | 4.2e-5 ± 5.7e-6 |
| Fibronectin-1 | FN1 | 6.1e-3 ± 5.8e-4 | **1.2e-2 ± 4.7e-4*** | 0.20 ± 3.3e-2 | 0.20 ± 9.0e-3 | 1.2e-2 ± 8.7e-4 | **2.9e-1 ± 8.9e-2*** | 3.1e-3 ± 8.6e-4 | 6.4e-3 ± 1.7e-3 |
| Fibroblast growth factor 23 | FGF23 | 1.2e-5 ± 2.4e-6 | 9.2e-5 ± 4.0e-5 | 6.2e-4 ± 3.4e-4 | 8.7e-4 ± 2.3e-4 | 5.6e-6 ± 1.8e-6 | 1.1e-5 ± 4.1e-6 | 2.0e-6 ± 7.6e-7 | 1.4e-6 ± 8.7e-7 |
| Connective Tissue Growth Factor | CNGF | 1.6e-2 ± 1.0e-3 | **3.0e-2 ± 8.2e-4*** | 4.1e-2 ± 2.3e-3 | 3.8e-2 ± 6.5e-3 | 9.1e-3 ± 5.9e-3 | **5.5e-2 ± 1.9e-2*** | 1.5e-3 ± 2.6e-4 | **2.4e-3 ± 1.2e-4*** |
| General transcription factor IIIA | GTF3A | 9.8e-4 ± 2.6e-4 | **1.4e-4 ± 4.2e-5*** | 7.6e-4 ± 1.9e-4 | 2.8e-4 ± 4.4e-5 | 3.5e-5 ± 7.8e-6 | 4.2e-5 ± 8.5e-6 | 2.0e-5 ± 1.1e-6 | 1.6e-5 ± 4.1e-6 |
| Integrin alpha-1 | ITGA1 | 3.6e-2 ± 2.2e-3 | 4.1e-2 ± 2.1e-3 | 6.4e-2 ± 9.4e-3 | 4.0e-2 ± 7.9e-3 | 3.8e-3 ± 2.8e-4 | **8.9e-3 ± 1.0e-3*** | 3.0e-3 ± 6.8e-5 | 2.6e-3 ± 3.7e-4 |
| Integrin alpha-2 | ITGA2 | 8.8e-3 ± 9.6e-4 | 9.9e-3 ± 6.6e-4 | 0.16 ± 4.9e-2 | 0.18 ± 1.9e-2 | 3.2e-4 ± 4.5e-5 | **8.9e-4 ± 2.4e-4*** | 4.6e-5 ± 6.2e-6 | 6.3e-5 ± 6.7e-6 |
| Growth differentiation factor 7 | GDF7 | 7.0e-4 ± 8.2e-5 | 6.3e-4 ± 2.2e-4 | 2.8e-3 ± 7.2e-4 | 2.1e-3 ± 3.5e-4 | 2.7e-5 ± 6.6e-6 | **1.7e-5 ± 4.2e-5*** | 1.1e-5 ± 3.0e-6 | 2.5e-5 ± 5.7e-6 |
| Integrin alpha-chain V | ITGAV | 7.0e-2 ± 7.3e-3 | 7.7e-2 ± 7.7e-3 | 0.25 ± 4.3e-2 | 0.27 ± 3.4e-2 | 2.7e-2 ± 2.9e-3 | **6.5e-2 ± 7.0e-3*** | 1.4e-2 ± 1.5e-3 | 1.9e-2 ± 2.9e-3 |
| Integrin beta-1 | ITGB1 | 7.7e-2 ± 5.6e-3 | 0.10 ± 6.7e-3 | 0.28 ± 3.5e-2 | 0.27 ± 3.1e-2 | 3.1e-2 ± 2.7e-3 | **0.12 ± 8.9e-3*** | 2.4e-3 ± 9.1e-4 | 3.4e-2 ± 5.2e-3 |
| Integrin beta-3 | ITGB3 | 1.3e-3 ± 2.1e-4 | **3.5e-3 ± 3.0e-4*** | 8.9e-3 ± 1.7e-3 | 7.3e-3 ± 6.0e-4 | 3.8e-4 ± 3.9e-5 | **3.8e-3 ± 2.5e-4*** | 1.7e-4 ± 3.0e-5 | **6.9e-4 ± 1.3e-4*** |
| Integrin beta-5 | ITGB5 | 3.7e-2 ± 2.2e-3 | 3.2e-2 ± 2.0e-3 | 8.0e-2 ± 1.9e-2 | 5.8e-2 ± 1.3e-2 | 4.0e-3 ± 4.4e-4 | **1.5e-2 ± 1.0e-3*** | 7.0e-3 ± 7.2e-4 | 1.0e-2 ± 1.5e-3 |
| Decorin (proteoglycan) | DCN | 1.3e-2 ± 1.9e-3 | 2.0e-2 ± 3.0e-3 | 0.47 ± 7.2e-2 | 0.38 ± 8.6e-2 | 6.3e-2 ± 6.1e-3 | **0.14 ± 1.5e-2*** | 2.6e-2 ± 5.4e-3 | 3.0e-2 ± 2.1e-3 |
| Beta cytoskeletal action | ACTB | 0.28 ± 2.0e-2 | 0.34 ± 3.5e-2 | 0.74 ± 9.0e-2 | 0.90 ± 9.9e-2 | 5.3e-2 ± 1.4e-2 | **0.16 ± 1.8e-2*** | 1.8e-2 ± 1.8e-3 | 3.8e-2 ± 8.9e-3 |
| Matrix metalloproteinase 1 (Collagenase 1) | MMP1 | 3.4e-5 ± 7.8e-6 | 6.5e-5 ± 8.2e-6 | 8.1e-5 ± 4.7e-6 | 1.3e-4 ± 3.4e-5 | 4.1e-5 ± 2.0e-5 | 7.0e-4 ± 5.8e-4 | 6.4e-6 ± 3.0e-6 | 6.7e-6 ± 2.6e-6 |
| Matrix metalloproteinase 2 (Gelatinase A) | MMP2 | 2.7e-3 ± 2.0e-4 | 3.2e-3 ± 3.2e-4 | 9.8e-3 ± 4.4e-3 | 6.6e-3 ± 4.2e-3 | 9.6e-3 ± 1.9e-3 | **3.7e-2 ± 3.4e-3*** | 2.5e-3 ± 4.9e-4 | 2.9e-3 ± 3.1e-4 |
| Matrix metalloproteinase 3 | MMP3 | 4.7e-5 ± 3.6e-6 | 7.6e-5 ± 1.0e-5 | 1.1e-4 ± 3.6e-5 | 1.1e-4 ± 3.3e-5 | 1.2e-4 ± 6.0e-5 | 9.1e-5 ± 3.6e-5 | 6.3e-6 ± 3.2e-6 | 5.6e-6 ± 1.6e-6 |
| Matrix metalloproteinase 8 | MMP8 | 2.6e-5 ± 5.8e-6 | **4.3e-4 ± 7.0e-5*** | 5.4e-5 ± 1.0e-5 | 1.9e-4 ± 5.5e-5 | 8.3e-6 ± 3.6e-6 | 3.4e-5 ± 1.4e-5 | 5.0e-6 ± 2.6e-6 | 3.2e-5 ± 1.8e-5 |
| Matrix metalloproteinase 9 (Gelatinase B) | MMP9 | 4.3e-4 ± 1.1e-4 | **1.3e-4 ± 3.4e-4*** | 7.4e-4 ± 3.1e-4 | 2.2e-3 ± 6.8e-4 | 7.2e-6 ± 7.3e-6 | **4.1e-5 ± 6.1e-6*** | 5.0e-6 ± 2.7e-6 | 2.4e-5 ± 1.4e-5 |
| Matrix metalloproteinase 13 | MMP13 | 1.4e-5 ± 5.1e-6 | **2.9e-4 ± 1.1e-4*** | 1.3e-4 ± 3.3e-5 | 4.7e-4 ± 2.4e-4 | 1.0e-6 ± 5.2e-7 | **1.1e-5 ± 4.2e-6*** | 9.0e-7 ± 5.1e-7 | 1.7e-6 ± 5.6e-7 |
| Tissue inhibitor of metalloproteinases 1 | TIMP1 | 5.7e-3 ± 1.2e-3 | **1.9e-3 ± 1.3e-3*** | 4.4e-2 ± 2.9e-3 | 5.4e-2 ± 1.6e-2 | 1.4e-3 ± 3.0e-4 | **2.3e-2 ± 2.3e-3*** | 8.1e-4 ± 2.1e-4 | 2.0e-3 ± 8.0e-4 |
| Tissue inhibitor of metalloproteinases 2 | TIMP2 | 9.2e-3 ± 8.7e-4 | 1.3e-2 ± 1.3e-3 | 4.8e-2 ± 5.2e-3 | 3.4e-2 ± 5.7e-3 | 1.1e-2 ± 1.1e-3 | **4.0e-2 ± 3.1e-3*** | 4.3e-3 ± 5.0e-4 | 4.8e-3 ± 2.3e-4 |
| Tissue inhibitor of metalloproteinases 3 | TIMP3 | 0.12 ± 1.4e-2 | 0.20 ± 2.7e-2 | 0.83 ± 0.21 | 1.19 ± 0.22 | 2.0e-2 ± 1.4e-3 | **2.7e-2 ± 1.9e-3*** | 1.0e-2 ± 7.6e-4 | 1.3e-2 ± 9.7e-4 |
| Transforming growth factor-beta 1 | TGFB1 | 4.7e-3 ± 5.8e-4 | **1.1e-2 ± 8.9e-4*** | 3.0e-2 ± 2.9e-3 | 2.5e-2 ± 3.0e-3 | 2.1e-3 ± 1.4e-4 | **8.0e-3 ± 7.3e-4*** | 2.0e-3 ± 1.3e-4 | **3.5e-3 ± 4.3e-4*** |
| Transforming growth factor-beta 2 | TGFB2 | 1.6e-3 ± 3.5e-5 | **2.0e-3 ± 8.3e-5*** | 7.5e-3 ± 1.4e-3 | 9.1e-3 ± 1.3e-3 | 6.6e-4 ± 6.5e-5 | **4.3e-3 ± 1.1e-3*** | 2.8e-4 ± 3.6e-5 | **4.5e-4 ± 4.4e-5*** |
| Transforming growth factor-beta 3 | TGFB3 | 4.0e-4 ± 7.9e-5 | 8.8e-4 ± 1.9e-4 | 2.1e-3 ± 1.1e-3 | 2.0e-3 ± 4.3e-4 | 5.0e-4 ± 1.0e-4 | **5.1e-3 ± 1.1e-3*** | 1.9e-3 ± 1.1e-4 | 1.7e-3 ± 2.7e-4 |
| Transforming growth factor-beta receptor 1 | TGFBR1 | 7.0e-3 ± 4.1e-4 | **1.1e-3 ± 9.0e-4*** | 2.7e-2 ± 1.8e-3 | 3.1e-2 ± 2.5e-3 | 1.2e-3 ± 1.2e-4 | **7.3e-3 ± 6.4e-4*** | 6.9e-4 ± 6.8e-5 | **1.4e-3 ± 1.6e-4*** |
| Transforming growth factor-beta receptor 2 | TGFBR2 | 1.9e-2 ± 1.5e-3 | 2.1e-2 ± 1.3e-3 | 8.5e-2 ± 1.1e-2 | 5.4e-2 ± 1.1e-2 | 3.6e-3 ± 7.5e-4 | **8.2e-3 ± 8.6e-4*** | 2.0e-3 ± 4.1e-4 | 2.1e-3 ± 2.6e-4 |
| Transforming growth factor-beta receptor 3 | TGFBR3 | 1.9e-2 ± 1.4e-3 | 2.5e-2 ± 1.4e-3 | 7.7e-2 ± 1.3e-2 | 6.3e-2 ± 8.4e-3 | 6.3e-3 ± 1.1e-3 | 6.5e-3 ± 6.1e-4 | 1.9e-3 ± 2.2e-4 | 2.1e-3 ± 1.4e-4 |
| Latent-Transforming growth factor beta binding protein-1 | LTBP1 | 5.6e-3 ± 3.7e-4 | 8.6e-3 ± 1.5e-3 | 0.15 ± 4.5e-2 | 0.15 ± 6.4e-2 | 2.7e-3 ± 3.2e-4 | **1.7e-2 ± 3.5e-3*** | 1.5e-3 ± 1.9e-4 | 2.2e-3 ± 3.8e-4 |
| SMAD family member 1 | SMAD1 | 5.7e-3 ± 3.7e-4 | 7.2e-3 ± 9.4e-5 | 3.2e-2 ± 3.5e-3 | 4.1e-2 ± 6.3e-3 | 2.3e-3 ± 2.0e-4 | **6.2e-3 ± 4.5e-4*** | 1.6e-3 ± 1.7e-4 | 2.3e-3 ± 4.6e-4 |
| SMAD family member 2 | SMAD2 | 1.1e-2 ± 1.1e-3 | 1.0e-2 ± 6.4e-4 | 4.3e-2 ± 5.1e-3 | 3.1e-2 ± 5.1e-3 | 2.6e-3 ± 3.0e-4 | **5.6e-3 ± 3.8e-4*** | 2.4e-3 ± 1.1e-4 | 2.6e-3 ± 2.9e-4 |
| SMAD family member 3 | SMAD3 | 1.6e-2 ± 1.5e-3 | 2.3e-2 ± 2.7e-3 | 3.5e-2 ± 5.6e-3 | **6.5e-2 ± 1.4e-2*** | 2.5e-3 ± 2.7e-4 | **5.8e-3 ± 4.0e-4*** | 2.0e-3 ± 1.7e-4 | **3.3e-3 ± 3.7e-4*** |
| SMAD family member 4 | SMAD4 | 2.3e-2 ± 1.3e-3 | 2.6e-2 ± 1.8e-3 | 5.1e-2 ± 5.1e-3 | 4.5e-2 ± 7.3e-3 | 5.5e-3 ± 5.1e-4 | **1.0e-2 ± 7.8e-4*** | 4.4e-3 ± 2.7e-4 | 5.2e-3 ± 5.5e-4 |
| SMAD family member 6 | SMAD6 | 3.3e-3 ± 4.6e-4 | 2.2e-3 ± 5.7e-4 | 8.9e-3 ± 2.3e-3 | 6.3e-3 ± 1.9e-3 | 5.0e-4 ± 4.5e-5 | **9.1e-4 ± 9.2e-5*** | 5.4e-4 ± 8.5e-5 | 4.2e-4 ± 8.6e-5 |
| SMAD family member 7 | SMAD7 | 7.1e-3 ± 1.2e-3 | 1.1e-2 ± 1.7e-3 | 1.7e-2 ± 3.9e-3 | 2.6e-2 ± 8.2e-3 | 1.1e-3 ± 1.9e-4 | **4.6e-3 ± 8.4e-4*** | 6.5e-4 ± 5.2e-5 | **1.0e-3 ± 9.5e-5*** |
| Bone morphogenetic protein 2 | BMP2 | 5.9e-3 ± 8.0e-4 | 6.9e-3 ± 2.9e-4 | 1.3e-2 ± 4.2e-3 | 1.4e-2 ± 2.5e-3 | 7.9e-4 ± 8.2e-5 | **1.8e-3 ± 1.8e-4*** | 6.3e-4 ± 7.2e-5 | 6.0e-4 ± 1.0e-4 |
| Bone morphogenetic protein 4 | BMP4 | 8.9e-3 ± 9.7e-4 | **5.1e-3 ± 3.0e-4*** | 1.7e-2 ± 6.2e-3 | 4.5e-3 ± 9.6e-4 | 9.8e-4 ± 1.7e-4 | 1.5e-3 ± 2.5e-4 | 9.5e-4 ± 1.3e-4 | 4.5e-4 ± 2.3e-4 |
| Bone morphogenetic protein 7 | BMP7 | 1.2e-3 ± 2.4e-4 | 1.2e-3 ± 4.0e-4 | 2.4e-5 ± 4.4e-6 | 2.4e-2 ± 8.2e-3 | 2.4e-5 ± 4.4e-6 | 8.5e-5 ± 3.0e-5 | 5.4e-6 ± 1.2e-6 | 4.0e-6 ± 8.2e-7 |

Data are expressed as mean ± SEM. *p<0.05 vs normals.

**Supplemental Table 3. Fold changes of gene expressions from normals**

| **Gene Name** | **Symbol** | **KC** | **KM** | **LA** | **LV** |
| --- | --- | --- | --- | --- | --- |
| ***Inflammatory cytokines and other growth factors*** |  |  |  |  |  |
| Tumor Necrosis Factor-alpha | TNF | **3.6*** | 1.8 | **22.0*** | **3.5*** |
| Interleukin-6 | IL6 | **24.7*** | 2.5 | **293.7*** | **40.0*** |
| Glycoprotein 130 | IL6ST | **1.8*** | 1.2 | **3.1*** | **1.8*** |
| Leukemia inhibitory factor | LIF | **23.0*** | **42.4*** | **45.7*** | **3.8*** |
| Endothlin-1 | EDN1 | 3.4 | 1.4 | **9.6*** | 1.3 |
| Monocyte chemoattractant protein-1, MCP-1 | CCL2 | **17.0*** | **10.4*** | **143.8*** | **27.7*** |
| Chemokine receptor 2 | CCR2 | 0.8 | 0.4 | 4.5 | 1.2 |
| Interleukin-1 beta | IL1B | **4.4*** | 0.5 | **10.2*** | 4.9 |
| Interleukin-10 | IL10 | 0.9 | 0.4 | **4.3*** | 0.5 |
| Interleukin-13 | IL13 | 1.4 | 0.8 | **0.9*** | 1.6 |
| Interferon-gamma | IFNG | **4.1*** | **4.7*** | **21.9*** | **6.3*** |
| Nerve growth factor receptor | NGFR | 1.2 | 0.8 | **2.4*** | 1.1 |
| Colony stimulating factor 3 | CSF3 | **7.4*** | **2.7*** | **133.6*** | **48.8*** |
| Hepatocyte growth factor | HGF | 1.5 | 0.5 | 2.5 | 0.8 |
| Epidermal growth factor | EGF | 1.0 | 0.1 | **3.7*** | 0.8 |
| Platelet-Derived Growth Factor A | PDGFA | 1.1 | 0.8 | **2.2*** | **1.4*** |
| Platelet-Derived Growth Factor B | PDGFB | **11.7*** | 2.4 | **2.3*** | 0.8 |
| Platelet-Derived Growth Factor Receptor B | PDGFRB | **1.7*** | 0.8 | **2.9*** | 1.3 |
| Vascular endothelial growth factor A | VEGFA | **1.8*** | 0.7 | 1.0 | 0.9 |
| Vascular cell adhesion molecule-1 | VCAM1 | **37.2*** | **4.3*** | **33.7*** | **10.4*** |
| Ras homolog gene family member A | RHOA | 1.1 | 1.2 | **2.9*** | 1.3 |
| Rho-associated coiled-coil containing protein-1 | ROCK1 | 1.1 | 0.9 | **2.8*** | 1.3 |
| Rho-associated coiled-coil containing protein-2 | ROCK2 | 1.2 | 1.2 | **2.3*** | **1.5*** |
| C-reactive protein | CRP | **7.1*** | 1.3 | 0.0 | 3.2 |
| Insulin | INS | 1.3 | 1.0 | **7.0*** | 2.8 |
| Von Willebrand factor | VWF | 1.0 | 0.6 | **4.3*** | 1.3 |
| Tissue plasminogen activator | PLAT | **3.9*** | **1.8*** | **13.5*** | **3.1*** |
| Plasminogen | PLG | 1.2 | 1.2 | 0.7 | 1.3 |
| Plasminogen activator inhibitor-1 | SERPINE1 | **26.5*** | **12.4*** | **86.7*** | **28.9*** |
| Adiponectin | ADIPOQ | 0.8 | 17.9 | 0.4 | 0.1 |
| Mitogen-activated protein kinase 1 | MAPK1 | 1.1 | 0.9 | **2.2*** | 1.1 |
| RAC-alpha serine/threonine-protein kinase 1 | AKT1 | 1.1 | 1.0 | **2.5*** | 1.2 |
| Signal transducer and activator of transcription 1 | STAT1 | 1.5 | 0.9 | 2.3 | 1.1 |
| Signal transducer and activator of transcription 3 | STAT3 | **2.0*** | 1.5 | **2.7*** | 1.5 |
| Nuclear factor kappa-B | NFKB1 | **5.5*** | **2.5*** | **7.2*** | **4.0*** |
| Transcription factor Sp1 | SP1 | 1.0 | 0.7 | **1.6*** | 1.0 |
| ***Renal inflammation and injury*** |  |  |  |  |  |
| Interleukin-1 alpha | IL1A | **7.2*** | **3.4*** | - | - |
| Interleukin-7 | IL7 | 1.0 | 1.1 | - | - |
| Interleukin-8 | IL8 | **38.4*** | **15.6*** | - | - |
| Interleukin-18 | IL18 | **3.9*** | 1.5 | - | - |
| Granulocyte Macrophage colony-stimulating Factor | CSF2 | 1.4 | 2.5 | - | - |
| granulocyte-colony stimulating factor | CSF3 | **8.5*** | 3.0 | - | - |
| Insulin-like growth factor 1 | IGF1 | 0.8 | 0.5 | - | - |
| Neural cell adhesion molecule 1 | NCAM1 | 1.3 | 0.7 | - | - |
| Intercellular Adhesion Molecule 1 | ICAM1 | **55.9*** | **14.7*** | - | - |
| P-selectin | SELP | **2.8*** | 0.8 | - | - |
| E-selectin | SELE | **139.0*** | **53.6*** | - | - |
| Chemokine (C-C motif) ligand 5, RANTES | CCL5 | 3.0 | 0.3 | - | - |
| C-C chemokine receptor type 1 | CCR1 | 1.8 | 1.1 | - | - |
| C-C chemokine receptor type 3 | CCR3 | 0.6 | 0.2 | - | - |
| Serum amyloid A1 | SAA1 | **13.8*** | 1.5 | - | - |
| N-acetyl-beta-(D)-glucosaminidase, NAG | MGEA5 | 1.1 | **2.2*** | - | - |
| kidney injury molecule-1, KIM-1 hepatitis A virus cellular receptor1 | HAVCR1 | 2.0 | 0.9 | - | - |
| Annexin A5 | ANXA5 | 0.1 | 0.6 | - | - |
| Thrombospondin 1, TSP-1 | THBS1 | 1.1 | 1.1 | - | - |
| Calponin 1 | CNN1 | **2.5*** | 1.1 | - | - |
| Fibulin-1 | FBLN1 | 1.4 | 0.8 | - | - |
| Ankyrin1 | ANK1 | 0.9 | 0.4 | - | - |
| Spectrin1 | SPTA1 | 1.1 | 0.9 | - | - |
| Heat shock protein 27 | HSPB1 | 1.6 | 1.3 | - | - |
| Toll-like receptor 2 | TLR2 | 1.2 | 0.7 | - | - |
| S100 calcium-binding protein A4 | S100A4 | 1.4 | 0.3 | - | - |
| S100 calcium-binding protein A6 | S100A6 | 1.1 | 1.3 | - | - |
| Clusterin, apolipoprotein J | CLU | 2.2 | 1.8 | - | - |
| Glutathione S-transferase alpha3 | GSTA3 | 0.4 | 3.5 | - | - |
| neutrophil gelatinase-associated lipoprotein  (NGAL), Lipocalin 2 (LCN2) | LCN2 | 1.0 | 0.9 | - | - |
| Trefoil factor 3 | TFF3 | 1.5 | 2.1 | - | - |
| FoxP3, forkhead box P3 | FOXP3 | 1.1 | 2.9 | - | - |
| Immediate early response 3 | IER3 | **8.6*** | **3.0*** | - | - |
| Signal transducer and activator of transcription 2 | STAT2 | **1.5*** | 0.7 | - | - |
| Matrix Gla protein | MGP | 1.9 | 1.1 | - | - |
| Superoxide dismutase 1 | SOD1 | 0.9 | 0.9 | - | - |
| Catalase | CAT | 0.9 | 0.4 | - | - |
| Na, K-ATPase1 | ATP1A1 | 1.3 | 0.4 | - | - |
| Na, K-ATPase2 | ATP1A2 | 1.3 | 1.4 | - | - |
| Lysyl oxidase | LOX | 1.1 | 0.4 | - | - |
| Kruppel-like transcription factor, Zf9 | KLF6 | **3.5*** | **2.4*** | - | - |
| C4 component | C4A | 1.4 | 2.7 | - | - |
| ***Apoptosis*** |  |  |  |  |  |
| Cytochrome c-1 | CYC1 | 1.0 | 0.5 | 1.0 | 0.8 |
| Apoptotic protease activating factor 1 | APAF1 | 0.8 | **0.8*** | 2.1* | 1.1 |
| B-cell lymphoma 2 | BCL2 | 1.2 | 1.1 | 1.9 | 1.4 |
| B-cell lymphoma 2 associated X protein | BAX | 1.0 | 0.7 | 2.0 | 1.0 |
| Bcl-2 associated death promoter | BAD | 1.0 | 0.6 | 0.7 | 0.9 |
| BCL2-antagonist/killer 1 | BAK1 | 1.0 | 1.1 | 2.4 | 1.0 |
| BH3 interacting-domain death agonist | BID | 1.0 | 0.7 | 1.4 | 1.1 |
| Baculoviral IAP repeat containing 2 | BIRC2 | **4.3*** | **2.2*** | **3.0*** | **1.8*** |
| Baculoviral IAP repeat containing 3 | BIRC3 | **25.9*** | **14.9*** | **25.2*** | **15.9*** |
| Caspase 2 | CASP2 | 1.4 | **1.5*** | **2.2*** | 1.4 |
| Caspase 3 | CASP3 | 1.1 | 0.7 | **2.6*** | 1.6 |
| Caspase 7 | CASP7 | 1.1 | 1.4 | **2.4*** | 1.7 |
| Caspase 8 | CASP8 | 0.9 | 1.0 | 2.1 | 2.6 |
| Caspase 9 | CASP9 | 1.0 | 0.9 | **2.2*** | 1.0 |
| CASP and FADD like apoptosis regulator | CFLAR | **2.0*** | 1.8 | **2.6*** | 1.8 |
| Conserved helix-loop-helix ubiquitous kinase | CHUK | 1.1 | 1.1 | 1.8 | 1.5 |
| Death-associated protein 6 | DAXX | 0.8 | 0.9 | 1.4 | 1.1 |
| Diablo homolog, mitochondrial | DIABLO | 1.1 | 1.3 | 1.9 | 1.3 |
| Calcineruin A alpha | PPP3CA | 1.2 | 0.6 | **2.8*** | 1.1 |
| Fas (TNF superfamily member 6) | FAS | **4.2*** | 1.7 | **8.1*** | **2.8*** |
| Fas Ligand | FASLG | 1.6 | 1.1 | **4.3*** | 2.0 |
| TNF receptor superfamily member 12A | TNFRSF12A | **4.6*** | **7.1*** | **11.0*** | 1.5 |
| TNF receptor superfamily member 1A | TNFRSF1A | **2.3*** | 1.5 | **3.2*** | 1.6 |
| TNF receptor superfamily member 21 | TNFRSF21 | 1.3 | 1.0 | 2.0 | 1.2 |
| TNF receptor superfamily member 25 | TNFRSF25 | 0.6 | 0.6 | 2.2 | 0.7 |
| TNF superfamily member 10 | TNFSF10 | **2.9*** | 1.3 | 1.6 | 0.9 |
| lymphotoxin alpha (TNF superfamily member1) | LTA | **4.3*** | 1.5 | 9.0 | 3.7 |
| Inhibitor of kappa light polypeptide B gene enhancer in B-cells, kinase beta | IKBKB | **3.7*** | 2.0 | **2.9*** | **2.4*** |
| Inhibitor of kappa light polypeptide G gene enhancer in B-cells,kinase gamma | IKBKG | 0.9 | 0.8 | 1.6 | 1.0 |
| Apoptosis signal-regulating kinase-1  (ASK1=MAP3K5) | MAP3K5 | 1.6 | **1.5*** | **2.5*** | 1.2 |
| Mitogen-activated protein kinase 8 | MAPK8 | 1.2 | 1.3 | 1.7 | 1.3 |
| Mitogen-activated protein kinase 9 | MAPK9 | 1.0 | 0.7 | **1.6*** | 1.0 |
| Nucleotide-binding oligomerization domain  containing protein 1 | NOD1 | 1.0 | 0.6 | **3.9*** | 1.3 |
| Myc | MYC | **7.7*** | **3.4*** | **4.7*** | **3.3*** |
| Nerve growth factor | NGF | 1.5 | 0.6 | 1.1 | 0.7 |
| Nerve growth factor receptor | NGFR | 1.4 | 0.9 | **2.6*** | 1.0 |
| Poly (ADF-ribose) polumerase 1 | PARP1 | 0.8 | **0.6*** | 1.4 | 1.0 |
| Transcription factor p65 | RELA | **2.3*** | **2.0*** | **3.1*** | **2.2*** |
| Calpain-2 | CAPN2 | 1.1 | 0.9 | **2.9*** | 1.4 |
| X-linked inhibitor of apoptosis | XIAP | 1.1 | 0.8 | 1.5 | 1.1 |
| Tumor protein p53 | TP53 | **2.2*** | 1.5 | **3.5*** | **2.3*** |
| Mitochondrial carnitine/acylcarnitine  carrier protein | SLC25A29 | **2.0*** | 1.2 | **2.8*** | 2.0 |
| ***Fibrosis*** |  |  |  |  |  |
| Collagen type I | COL1A1 | **1.8*** | 0.4 | **60.6*** | 2.2 |
| Collagen type III | COL3A1 | 1.0 | 0.5 | **16.0*** | 1.6 |
| Collagen type IV | COL4A1 | **1.7*** | 1.1 | **9.3*** | 20.9 |
| Collagen type IV | COL4A2 | **1.5*** | 0.9 | **8.9*** | 1.8 |
| Collagen type XV | COL15A1 | 1.3 | 0.3 | **3.5*** | 1.4 |
| Contactin-1 | CNTN1 | 1.5 | 0.3 | 0.7 | 1.5 |
| Fibronectin-1 | FN1 | **1.9*** | 1.0 | **24.2*** | 2.0 |
| Fibroblast growth factor 23 | FGF23 | 7.7 | 1.4 | 2.0 | 0.7 |
| Connective Tissue Growth Factor | CNGF | **1.9*** | 0.9 | **6.0*** | **1.5*** |
| General transcription factor IIIA | GTF3A | **0.1*** | 0.4 | 1.2 | 0.8 |
| Integrin alpha-1 | ITGA1 | 1.2 | 0.6 | **2.3*** | 0.9 |
| Integrin alpha-2 | ITGA2 | 1.1 | 1.1 | **2.8*** | 1.4 |
| Growth differentiation factor 7 | GDF7 | 0.9 | 0.7 | **0.6*** | 2.3 |
| Integrin alpha-chain V | ITGAV | 1.1 | 1.1 | **2.4*** | 1.4 |
| Integrin beta-1 | ITGB1 | 1.3 | 1.0 | **3.9*** | 14.3 |
| Integrin beta-3 | ITGB3 | **2.8*** | 0.8 | **10.1*** | **4.1*** |
| Integrin beta-5 | ITGB5 | 0.9 | 0.7 | **3.8*** | 1.4 |
| Decorin (proteoglycan) | DCN | 1.6 | 0.8 | **2.3*** | 1.2 |
| Beta cytoskeletal action | ACTB | 1.2 | 1.2 | **3.0*** | 2.1 |
| Matrix metalloproteinase 1 (Collagenase 1) | MMP1 | 1.9 | 1.6 | 17.1 | 1.0 |
| Matrix metalloproteinase 2 (Gelatinase A) | MMP2 | 1.2 | 0.7 | **3.9*** | 1.1 |
| Matrix metalloproteinase 3 | MMP3 | 1.6 | 1.1 | 0.8 | 0.9 |
| Matrix metalloproteinase 8 | MMP8 | **16.5*** | 3.6 | 4.1 | 6.4 |
| Matrix metalloproteinase 9 (Gelatinase B) | MMP9 | **0.3*** | 3.0 | **5.7*** | 4.8 |
| Matrix metalloproteinase 13 | MMP13 | **20.6*** | 3.6 | **10.6*** | 1.9 |
| Tissue inhibitor of metalloproteinases 1 | TIMP1 | **0.3*** | 1.2 | **15.9*** | 2.5 |
| Tissue inhibitor of metalloproteinases 2 | TIMP2 | 1.5 | 0.7 | **3.6*** | 1.1 |
| Tissue inhibitor of metalloproteinases 3 | TIMP3 | 1.6 | 1.4 | **1.4*** | 1.2 |
| Transforming growth factor-beta 1 | TGFB1 | **2.3*** | 0.8 | **3.9*** | **1.8*** |
| Transforming growth factor-beta 2 | TGFB2 | **1.2*** | 1.2 | **6.6*** | **1.6*** |
| Transforming growth factor-beta 3 | TGFB3 | 2.2 | 0.9 | **10.2*** | 0.9 |
| Transforming growth factor-beta receptor 1 | TGFBR1 | **0.2*** | 1.2 | **6.3*** | **2.0*** |
| Transforming growth factor-beta receptor 2 | TGFBR2 | 1.1 | 0.6 | **2.3*** | 1.1 |
| Transforming growth factor-beta receptor 3 | TGFBR3 | 1.3 | 0.8 | 1.0 | 1.1 |
| Latent-Transforming growth factor beta binding protein-1 | LTBP1 | 1.5 | 1.0 | **6.5*** | 1.5 |
| SMAD family member 1 | SMAD1 | 1.3 | 1.3 | **2.6*** | 1.4 |
| SMAD family member 2 | SMAD2 | 0.9 | 0.7 | **2.2*** | 1.1 |
| SMAD family member 3 | SMAD3 | 1.4 | **1.9*** | **2.3*** | **1.7*** |
| SMAD family member 4 | SMAD4 | 1.2 | 0.9 | **1.9*** | 1.2 |
| SMAD family member 6 | SMAD6 | 0.7 | 0.7 | **1.8*** | 0.8 |
| SMAD family member 7 | SMAD7 | 1.5 | 1.5 | **4.2*** | **1.6*** |
| Bone morphogenetic protein 2 | BMP2 | 1.2 | 1.1 | **2.3*** | 1.0 |
| Bone morphogenetic protein 4 | BMP4 | **0.6*** | 0.3 | 1.5 | 0.5 |
| Bone morphogenetic protein 7 | BMP7 | 1.0 | 1.0 | 3.5 | 0.7 |

Data are expressed as fold changes from normals in each organ. *p<0.05 vs normals.
